# Prognosis of clear cell renal cell carcinoma (ccRCC) based on a six-lncRNA-based risk score: an investigation based on RNA-sequencing data

**DOI:** 10.1186/s12967-019-2032-y

**Published:** 2019-08-23

**Authors:** Jiang-hui Zeng, Wei Lu, Liang Liang, Gang Chen, Hui-hua Lan, Xiu-Yun Liang, Xu Zhu

**Affiliations:** 1 Department of Clinical Laboratory, The Third Affiliated Hospital of Guangxi Medical University/Nanning Second People’s Hospital, 13 Dancun Road, Nanning, 530031 Guangxi Zhuang Autonomous Region People’s Republic of China; 2 Department of Pathology, The Third Affiliated Hospital of Guangxi Medical University/Nanning Second People’s Hospital, 13 Dancun Road, Nanning, 530031 Guangxi Zhuang Autonomous Region People’s Republic of China; 3 Department of General Surgery, The Second Affiliated Hospital of Guangxi Medical University, 166 Daxuedong Road, Nanning, Guangxi Zhuang Autonomous Region People’s Republic of China; 4 Department of Pathology, First Affiliated Hospital of Guangxi Medical University, 6 Shuangyong Road, Nanning, 530021 Guangxi Zhuang Autonomous Region People’s Republic of China; 5 Department of Clinical Laboratory, The People’s Hospital of Guangxi Zhuang Autonomous Region, 6 Taoyuan Road, Nanning, Guangxi Zhuang Autonomous Region People’s Republic of China

**Keywords:** lncRNA, Clear cell renal cell carcinoma, Prognosis

## Abstract

**Background:**

The scientific understanding of long non-coding RNAs (lncRNAs) has improved in recent decades. Nevertheless, there has been little research into the role that lncRNAs play in clear cell renal cell carcinoma (ccRCC). More lncRNAs are assumed to influence the progression of ccRCC via their own molecular mechanisms.

**Methods:**

This study investigated the prognostic significance of differentially expressed lncRNAs by mining high-throughput lncRNA-sequencing data from The Cancer Genome Atlas (TCGA) containing 13,198 lncRNAs from 539 patients. Differentially expressed lncRNAs were assessed using the R packages edgeR and DESeq. The prognostic significance of lncRNAs was measured using univariate Cox proportional hazards regression. ccRCC patients were then categorized into high- and low-score cohorts based on the cumulative distribution curve inflection point the of risk score, which was generated by the multivariate Cox regression model. Samples from the TCGA dataset were divided into training and validation subsets to verify the prognostic risk model. Bioinformatics methods, gene set enrichment analysis, and protein–protein interaction networks, Gene Ontology, and Kyoto Encyclopedia of Genes and Genomes analyses were subsequently used.

**Results:**

It was found that the risk score based on 6 novel lncRNAs (CTA-384D8.35, CTD-2263F21.1, LINC01510, RP11-352G9.1, RP11-395B7.2, RP11-426C22.4) exhibited superior prognostic value for ccRCC. Moreover, we categorized the cases into two groups (high-risk and low-risk), and also examined related pathways and genetic differences between them. Kaplan–Meier curves indicated that the median survival time of patients in the high-risk group was 73.5 months, much shorter than that of the low-risk group (112.6 months; *P* < 0.05). Furthermore, the risk score predicted the 5-year survival of all 539 ccRCC patients (AUC at 5 years, 0.683; concordance index [C-index], 0.853; 95% CI 0.817–0.889). The training set and validation set also showed similar performance (AUC at 5 years, 0.649 and 0.681, respectively; C-index, 0.822 and 0.891; 95% CI 0.774–0.870 and 0.844–0.938).

**Conclusions:**

The results of this study can be applied to analyzing various prognostic factors, leading to new possibilities for clinical diagnosis and prognosis of ccRCC.

## Background

The role of genomes in biological processes has become better understood in recent decades, as researchers have gradually come to recognize the roles of individual transcripts in particular. New high-throughput sequencing technologies have enabled the detection of novel transcripts through increased sensitivity. These recent advances have facilitated more comprehensive and more thorough research into the effects of transcription and translation [1–3]. At present, much is understood about messenger RNAs and other RNAs, including transfer RNAs, small nuclear RNAs, small nucleolar RNAs, and micro RNAs, but the roles, types, and biological significance of long non-coding RNAs (lncRNAs) have yet to be elucidated [4–6].

Kidney cancer is one of the most prevalent urinary tract cancers in adults. In the United States, a total of 63,900 new cases of kidney and renal pelvis cancers were projected (40,610 and 23,380 for male and female patients, respectively), with an estimated 14,400 deaths (9470 and 4930 for males and females, respectively) in 2017 [7]. With approximately 3% mortality for all cases, the rate continues to soar [7]. In China, 66,800 cases of kidney cancer were newly diagnosed, with a 2.34% mortality rate in 2015 [8]. Histologically, clear cell renal cell carcinoma (ccRCC) is the most widespread kidney cancer subtype, constituting 70% of kidney cancers, followed by kidney renal papillary cell carcinoma (10%) and chromophobe renal cell carcinoma (5%) [9–11].

Recently, lncRNAs have been revealed to play a role in tumorigenesis, disease development, and metastasis in ccRCC, in both oncogenic and tumor-suppressing roles that modulate a number of biological and pathological processes [12–17]. Nevertheless, scant prognosis-related research has been conducted on lncRNAs in ccRCC, and more lncRNAs are assumed to influence ccRCC progression via their own molecular mechanisms. Thus, the present study aimed to investigate the prognostic significance of differentially-expressed lncRNAs by mining high-throughput RNA-sequencing data from The Cancer Genome Atlas (TCGA). A risk score based on 6 novel lncRNAs exhibited superior prognostic value for ccRCC outcomes.

## Methods

### Patient cohort from TCGA dataset

RNA sequencing (RNA-Seq) raw counts data (level 3) from ccRCC patients, which were generated using the Illumina HiSeq RNASeq platform, were obtained from the TCGA data portal (https://tcga-data.nci.nih.gov/tcga/). These data corresponded to 539 ccRCC tissues and 72 adjacent non-tumorous renal tissue samples deposited on or before May 31, 2017. The ultimate status of the ccRCC patients in our study was captured as overall survival (OS) data. The average follow-up period was 44.9 months. The data were retrieved from TCGA, which is a community resource project offering data for research; approval from the local ethics committee was not necessary for the current study, as it complied with TCGA publication principles and data use policies.

### Assessment of differentially expressed lncRNAs

The ccRCC RNA-Seq data contained 60,483 messenger RNAs, including 13,198 lncRNAs that have been labelled in NCBI (https://www.ncbi.nlm.nih.gov/) or GENCODE databases (http://www.gencodegenes.org/). Differentially expressed lncRNAs were assessed using edgeR and DESeq packages for the R statistical computing environment (using adjusted *P* < 0.05 and |log_2_FC| > 2 thresholds, respectively) [18, 19]. The expression level of each lncRNA was assessed using DESeq. The lncRNA expression data were displayed as log_2_-transformation. The final candidate lncRNAs were determined using the two R packages. Student’s *t*-tests (SPSS 22.0, IBM Corp., Armonk, NY) were employed to assess differential expression of the 6 candidate lncRNAs for discriminating between ccRCC and non-cancerous kidney tissues.

### ccRCC prognosis capabilities based on differentially expressed lncRNAs

The differentially expressed lncRNAs for which relative expression levels were below 1 in more than 10% of all subjects were eliminated from subsequent analyses. Similarly, lncRNAs were excluded if they lacked adequate clinical information. The final prognostic analysis included a total of 530 samples with expression data for 370 lncRNAs. Samples from the TCGA dataset were divided into training and validation sets, which were randomly selected from 530 tumor samples to verify the prognostic risk model.

The prognostic significance of lncRNAs was primarily measured by univariate Cox proportional hazard regression (*P* < 0.01). Statistically significant indicators, including lncRNAs, were further confirmed via multivariate Cox stepwise regression. Furthermore, the relationships between the expression of these 6 lncRNAs and various clinicopathological features were assessed by Student’s *t*-tests and Spearman correlation analysis.

### Clinical role of the risk score generated by the key lncRNAs

An lncRNA-based prognosis risk score was generated from a linear combination of the expression level multiplied by the regression coefficient acquired from the multivariate Cox regression model (β) with the following formula as previously reported [20, 21]:

$${\text{Risk score }} = \mathop \sum \limits_{n = 1}^{\infty } \left( {e_{n} \;*\;\beta_{n} } \right)$$

The β value is the estimated regression coefficient of the lncRNA derived from the multivariate Cox stepwise regression analysis and *e* indicates the expression profiles of the lncRNA.

Based on the cumulative distribution curve inflection point of the risk score, ccRCC patients were categorized into high- and low-score cohorts. Univariate and multivariate Cox proportional hazards regression analyses were conducted to further assess the efficacy of this prognostic risk score, and adjustments were made based on risk score, race, sex, age, tumor stage, distant metastasis, lymph node metastasis, neoplasmic cancer status, clinical stage, and tumor grade. Hazard ratios (HRs) with 95% confidence intervals (CIs) were examined. A time-dependent receiver operating characteristic (ROC) curve analysis within 5 years was also performed with the R package survival ROC in order to calculate the prognostic accuracy of the model for time-dependent disease outcomes. Kaplan–Meier (K–M) survival curves were assessed to determine correlations between all parameters (clinical aspects and six-lncRNA-based risk scores) and ccRCC patient OS. A concordance index (C-index) was used to measure the predictive accuracy and discriminative ability of the nomograms.

A ROC curve was used to assess the prognostic effectiveness of the six-lncRNA-based risk scores for clinical progress of ccRCC patients. A two-sided *P*-value < 0.05 threshold was used to assess corresponding results as statistically significant. SPSS 22.0 (IBM Corp.) was utilized for these statistical analyses.

### Different signaling pathways between high- and low-risk groups

Gene set enrichment analysis (GSEA) was carried out using GSEA software (http://www.broadinstitute.org/gsea) with the MSigDB C2 CP canonical pathways gene set collection [22–27]. A total of 60,483 genes were imported for GSEA. Gene sets with a nominal *P*-value less than 0.05 and a false discovery rate (FDR) value less than 0.25 were considered to be significantly enriched. For the most important pathways, protein–protein interaction (PPI) network analysis was also performed using the Search Tool for the Retrieval of Interacting Genes (STRING) database (http://www.string-db.org/) [28, 29]. Differentially expressed genes (DEGs) were identified using the edgeR package with *P*_adj_ < 0.01 and |log_2_FC| > 3 [30–33] between the high- and low-risk score groups for ccRCC and normal kidney samples. The DEG results were rendered as volcano plots and heatmaps. Identified DEGs were used to perform Gene Ontology (GO) and Kyoto Encyclopedia of Genes and Genomes (KEGG) analyses with the DAVID online tool (http://david.abcc.ncifcrf.gov/) [28, 29].

### Validation by Gene Expression Omnibus DataSets and International Cancer Genomics Consortium database

We collected the relevant microarrays from Gene Expression Omnibus (GEO) DataSets to validate the clinical roles of the six lncRNAs, the following search terms were used: (kidney OR nephridium OR renal) AND (“clear cell”) AND (cancer OR carcinoma OR tumor OR neoplas* OR malignan* OR adenocarcinoma OR ccRCC) [28, 34]. Differences in lncRNA expression levels between different groups were assessed using Student’s *t*-tests. Furthermore, we searched ccRCC dataset through the International Cancer Genomics Consortium (ICGC) database (https://icgc.org/) to verify to verify the effectiveness of prognostic model.

## Results

### Differentially expressed ccRCC lncRNAs

**Fig. 1:**
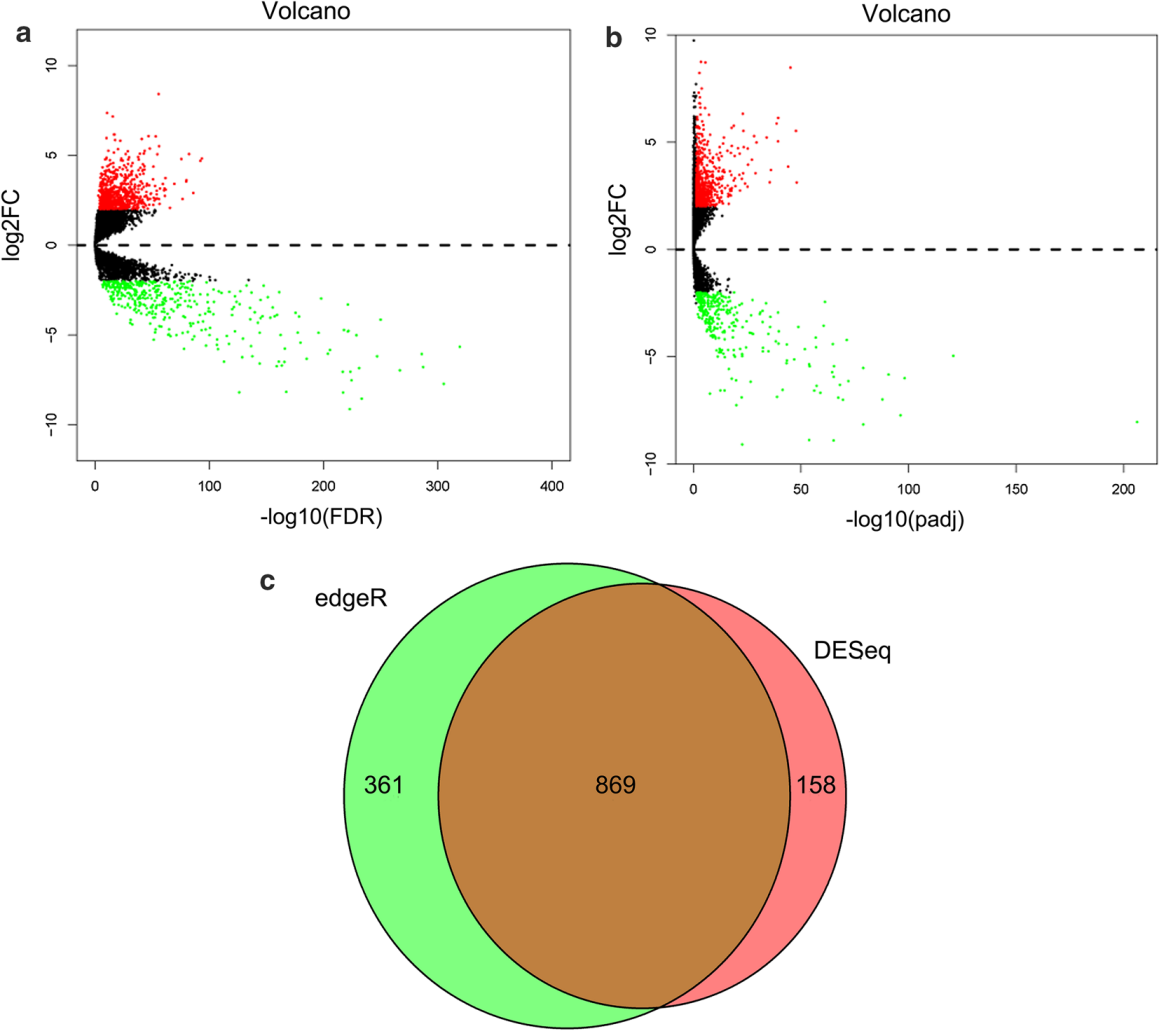
Differentially expressed lncRNAs analysis. **a** Differentially expressed lncRNAs identified using the edgeR package. Red and green points indicate upregulated and downregulated DELs, respectively (|log_2_FC| > 2). **b** Differentially expressed lncRNAs identified using the DESeq package. The individual datapoints are the same as those in **a** (|log_2_FC| > 2). **c** Overlapping differentially expressed lncRNAs

The analysis of 60,483 TCGA messenger RNAs revealed the differential expression of 13,198 lncRNAs based on the results of the R packages edgeR and DESeq. Significantly differentially expressed lncRNAs (*n* = 869) were obtained for subsequent prognostic analysis (Fig. 1). Among these 869 lncRNAs, 555 were upregulated and 314 were downregulated.

### Assessment of prognosis based on differentially-expressed lncRNAs

**Fig. 2:**
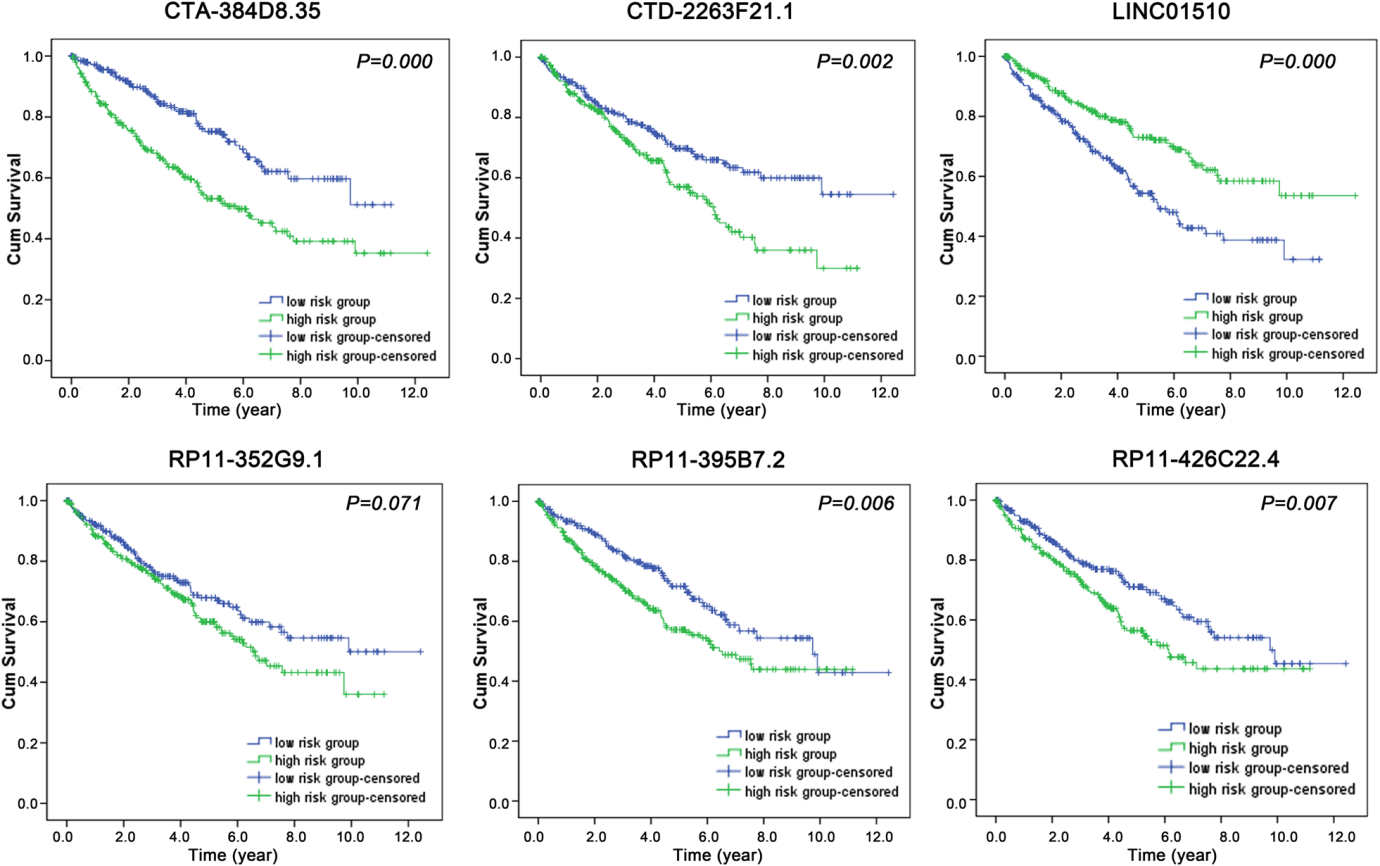
The independent prognostic features of these 6 lncRNAs. Survival analysis of these 6 lncRNAs was shown with Kaplan–Meier survival curves

**Fig. 3:**
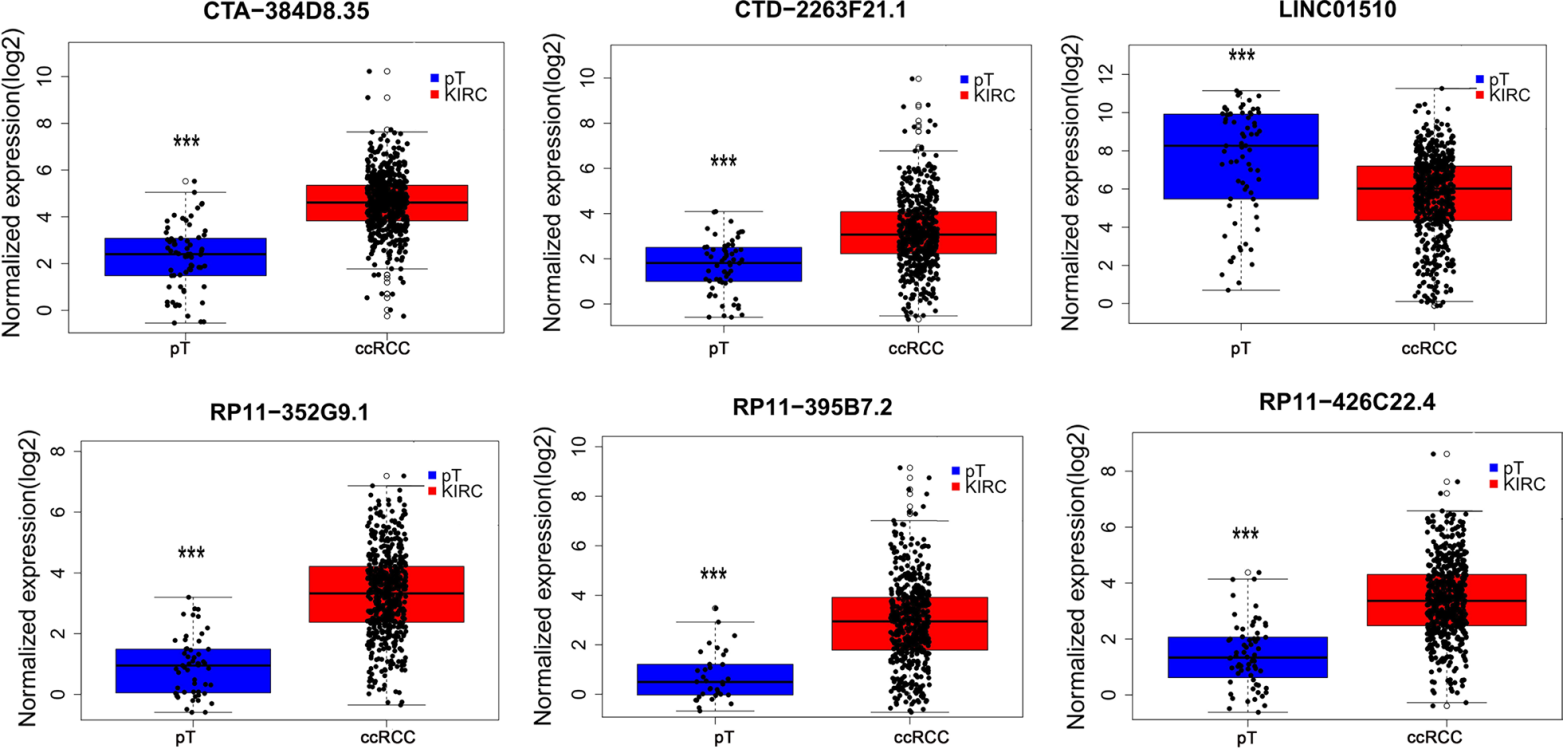
Differential expression of the six key lncRNAs between clear cell renal cell carcinoma (ccRCC) and para-tumorous (pT) renal tissues. *P < 0.05; **P < 0.01; ***P < 0.001

**Fig. 4:**
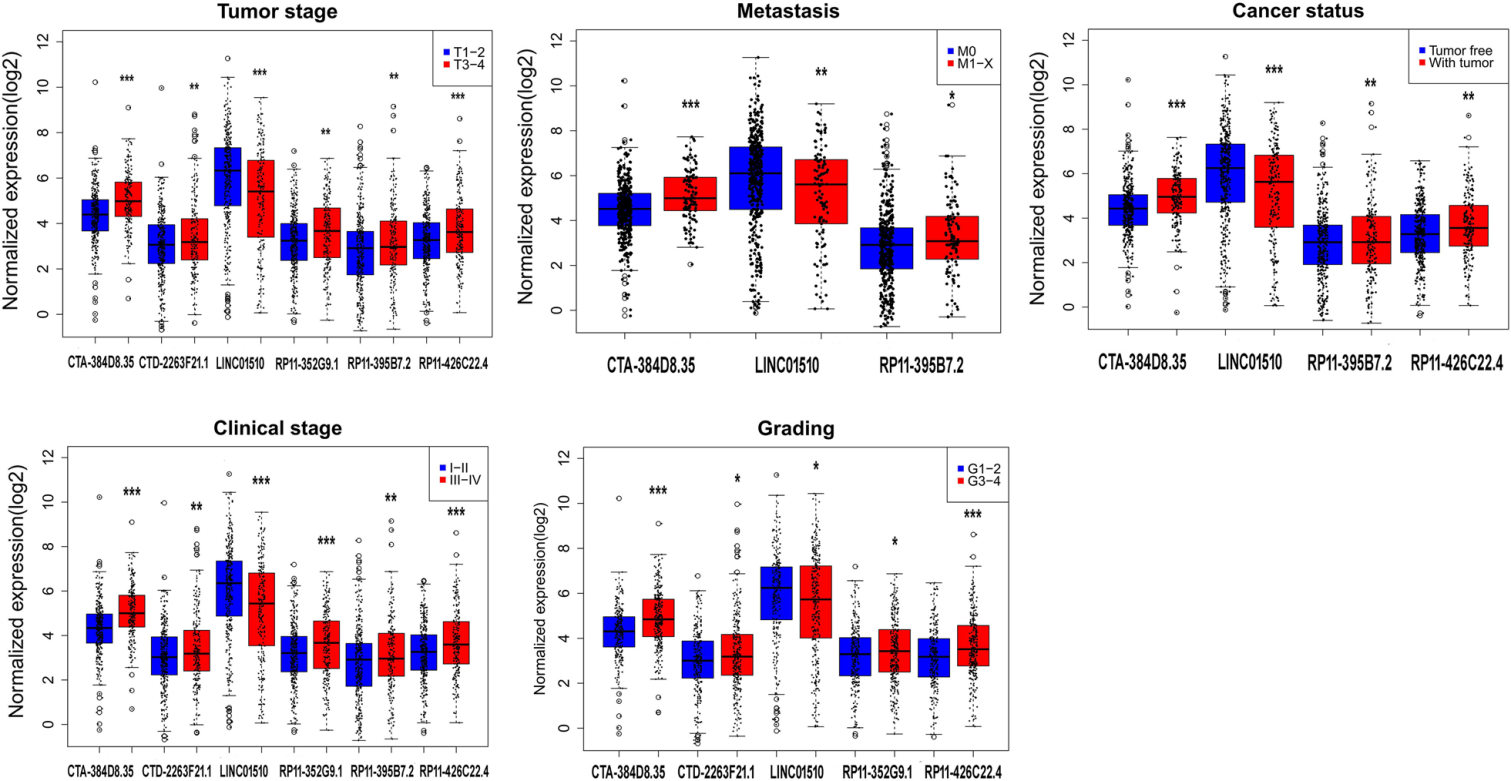
Association between the expression of key lncRNAs and clinicopathological features in clear cell renal cell carcinoma (ccRCC). Statistically significant differences in the expression of these key lncRNAs were associated with various clinicopathological features: tumor stage (T1/T2 vs. T3/T4), distant metastasis (M0 vs. M1–X), cancer status (tumor free vs. with tumor), clinical stage (I/II vs. III/IV), and grade. The different lncRNAs are arrayed along the *x*-axis, while the *y*-axis indicates normalized expression (log_2_). *P < 0.05; **P < 0.01; ***P < 0.001

**Fig. 5:**
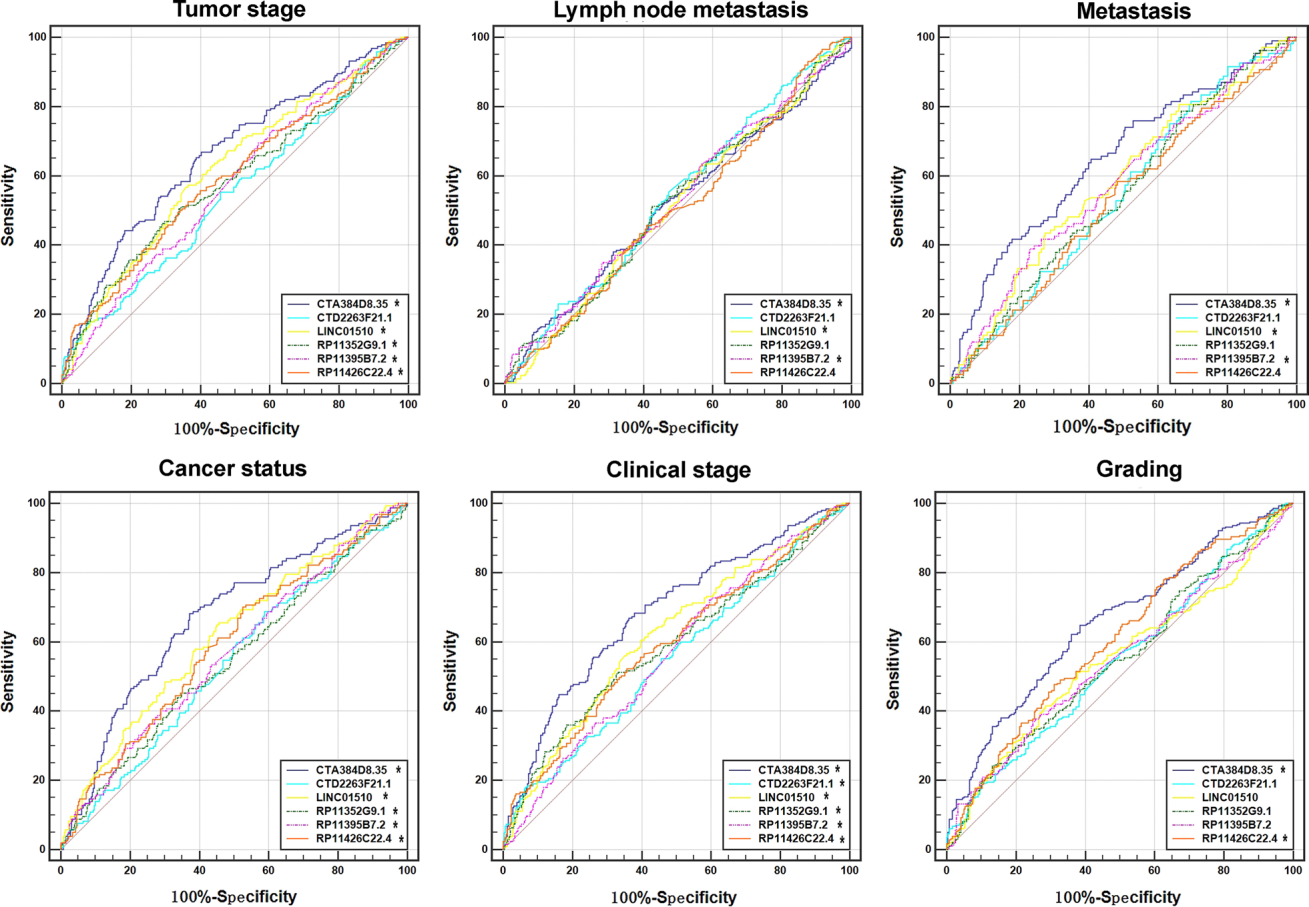
Predictive power of six key lncRNAs for clinical progression of clear cell renal cell carcinoma (ccRCC) using receiver operating characteristic (ROC) curves. ROC curves were constructed to evaluate the predicted value of each key lncRNA for cancer progression including advanced tumor stages (T3–4), lymph node metastasis, metastasis, cancer status (with tumor), higher clinical stages (III–IV), and grade (G3–4). The *x*-axis shows the false positive rate, presented as “100%-Specificity,” while the *y*-axis indicates the true positive rate, shown as “Sensitivity.” *P < 0.05 for AUC of each lncRNA

After eliminating the samples without adequate associated survival data, we identified 530 cases for diagnostic assessment. The lncRNAs lacking expression data in 10% of the samples were also excluded from the prognosis assessment. Using univariate Cox regression, we discovered that 107 lncRNAs in total displayed prognostic capabilities for ccRCC outcomes (*P* < 0.01). This conclusion was validated by multivariate Cox regression, and CTA-384D8.35, CTD-2263F21.1, LINC01510, RP11-352G9.1, RP11-395B7.2, and RP11-426C22.4 were confirmed to be independent prognostic biomarkers for ccRCC (Table 1 and Additional file 1: Table S1). In addition, the independent prognostic features of these 6 lncRNAs were shown in Fig. 2 using the Kaplan–Meier survival curves. The original expression differences of these 6 lncRNAs between ccRCC and non-cancerous kidney tissues were also evaluated. Remarkably higher expression levels were noted for CTA-384D8.35, CTD-2263F21.1, RP11-352G9.1, RP11-395B7.2, and RP11-426C22.4, while predominantly lower expression was observed for LINC01510 in ccRCC samples (Fig. 3). The association between the expression of the 6 identified lncRNAs and clinicopathological features were further analyzed by *t*-test. CTA-384D8.35 expression was related to tumor stage, metastasis, cancer status, clinical stage, and grade; CTD-2263F21.1 expression was related to tumor stage, clinical stage, and grade; LINC01510 expression was related to tumor stage, metastasis, cancer status, clinical stage, and grade; RP11-352G9.1 expression was related to tumor stage, cancer status, clinical stage, and grade; RP11-395B7.2 expression was related to tumor stage, metastasis, cancer status, and clinical stage; RP11-426C22.4 expression was related to tumor stage, cancer status, clinical stage, and grade (all *P* < 0.05). More importantly, as shown in Table 2 and Figs. 4 and 5, the levels of these 6 lncRNAs predicted the clinical progression of ccRCC.

**Table 1:** Detailed summary of six prognostic lncRNAs in clear cell renal cell carcinoma (ccRCC)

lncRNA	Esenble ID	Location	Log2 FC	β(Cox)	SE	P-value	Exp(B)	Lower	Upper
CTA-384D8.35	ENSG00000272666	Chromosome 22: 50,542,305–50,542,906	2.200229534	0.527	0.153	0.001	1.695	1.255	2.289
CTD-2263F21.1	ENSG00000251257	Chromosome 5: 38,460,925–38,468,339	2.101799115	0.238	0.11	0.031	1.268	1.022	1.573
LINC01510	ENSG00000231210	Chromosome 7: 116,570,960–116,614,820	− 2.298623575	− 0.304	0.078	< 0.001	0.738	0.634	0.859
RP11-352G9.1	ENSG00000273009	Chromosome 3: 195,913,078–195,913,683	2.68714083	0.459	0.144	0.001	1.583	1.194	2.097
RP11-395B7.2	ENSG00000274993	Chromosome 7: 100,963,828–100,968,124	3.610473993	0.25	0.107	0.02	1.284	1.04	1.584
RP11-426C22.4	ENSG00000259807	Chromosome 16: 29,217,170–29,220,031	2.061342232	− 0.309	0.124	0.012	0.734	0.576	0.935

**Table 2:** Association between six lncRNAs and clinical features of clear cell renal cell carcinoma (ccRCC) patients

Factor	CTA-384D8.35	CTD-2263F21.1	LINC01510	RP11-352G9.1	RP11-395B7.2	RP11-426C22.4
Tumor stage (T3–4/T1–2)						
*t*	6.217	2.587	− 4.611	3.190	2.853	4.004
*P*	*<* *0.001*	*0.010*	*<* *0.001*	*0.002*	*0.005*	*<* *0.001*
AUC (95% CIs)	0.663 (0.614, 0.712)	0.547 (0.495, 0.599)	0.383 (0.333, 0.433)	0.583 (0.531, 0.635)	0.572 (0.522, 0.623)	0.594 (0.542, 0.645)
*P*	*<* *0.001*	0.072	*<* *0.001*	*0.002*	*0.006*	< *0.001*
Lymph node metastasis (N1–NX/N0)						
*t*	0.023	− 1.612	0.090	− 0.865	1.370	− 0.567
*P*	0.981	0.108	0.928	0.387	0.171	0.571
AUC (95% CIs)	0.484 (0.435, 0.533)	0.464 (0.414, 0.513)	0.510 (0.460, 0.559)	0.482 (0.433, 0.532)	0.526 (0.477, 0.575)	0.497 (0.448, 0.547)
*P*	0.529	0.149	0.696	0.479	0.300	0.911
Metastasis (M1–MX/M0)						
*t*	4.969	1.338	− 2.637	1.699	2.475	0.685
*P*	< 0.001	0.181	0.009	0.090	0.014	0.494
AUC (95% CIs)	0.651 (0.592, 0.711)	0.547 (0.489, 0.605)	0.416 (0.357, 0.476)	0.550 (0.491, 0.609)	0.578 (0.517, 0.639)	0.530 (0.470, 0.591)
*P*	< 0.001	0.132	0.007	0.110	0.013	0.329
Cancer status (with tumor/tumor free)						
*t*	5.324	1.356	− 4.431	1.665	2.857	3.406
*P*	< 0.001	0.176	< 0.001	0.097	0.004	0.001
AUC (95%CIs)	0.669 (0.618, 0.721)	0.540 (0.486, 0.594)	0.383 (0.330, 0.436)	0.547 (0.492, 0.601)	0.569 (0.515, 0.623)	0.590 (0.536, 0.644)
*P*	< 0.001	0.151	< 0.001	0.094	0.013	0.001
Clinical stage (III–IV/I–II)						
*t*	7.074	2.840	− 4.624	3.511	2.694	4.051
*P*	< 0.001	0.005	< 0.001	< 0.001	0.007	< 0.001
AUC (95% CIs)	0.685 (0.638, 0.732)	0.556 ( 0.505, 0.607)	0.381 (0.332, 0.430)	0.589 (0.538, 0.640)	0.567 (0.518, 0.617)	0.594 (0.544, 0.645)
*P*	*<* *0.001*	*0.031*	*<* *0.001*	*0.001*	*0.009*	*<* *0.001*
Grade (G3–4/G1–2)						
*t*	6.241	2.555	− 2.324	2.091	1.963	4.258
*P*	*<* *0.001*	*0.011*	*0.021*	*0.037*	0.050	*<* *0.001*
AUC (95% CIs)	0.655 (0.608, 0.701)	0.545 (0.495, 0.594)	0.451 (0.402, 0.500)	0.55 (0.500, 0.599)	0.549 (0.500, 0.599)	0.604 (0.556, 0.653)
*P*	*<* *0.001*	0.078	0.053	0.051	0.051	*<* *0.001*

Italic represented the difference was statistically significant

### Clinical role of the six-lncRNA-based risk score

Next, the six-lncRNA-based risk score for predicting OS was calculated using a formula consisting of the expression level multiplied by the regression coefficient derived from the multivariate Cox regression model (β) values:

$$\begin{aligned} {\text{Risk}}\;{\text{score}} & = 0.527 \times e_{{{\text{CTA{-}}}384{\text{D}}8.35}} + 0.238\times e_{{{\text{CTD{-}2263F21}}. 1}} - 0. 30 4 { } \times e_{{{\text{LINC}}0 1 5 10}} \hfill \\ & \quad + 0. 4 5 9 { } \times e_{{{\text{RP11{-}352G9}}. 1}} + 0.25 { } \times e_{{{\text{RP11{-}395B7}}.2}} - 0. 30 9\times e_{{{\text{RP11{-}426C22}}.4}} \hfill \\ \end{aligned}$$

**Fig. 6:**
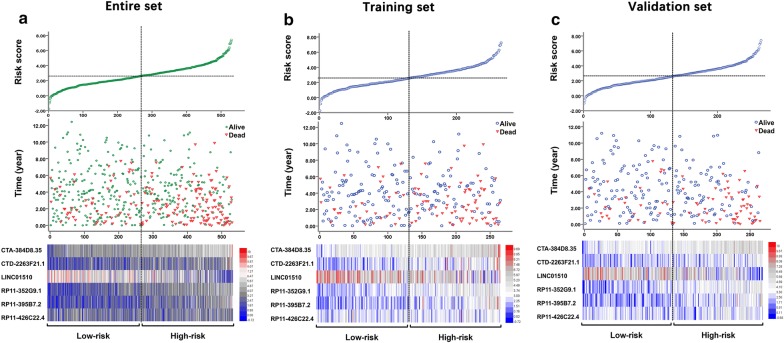
Analysis of lncRNA risk score in clear cell renal cell carcinoma (ccRCC) patients. **a** The entire set (530 tumor samples). **b** The training set (265 tumor samples). **c** The validation set (265 tumor samples). Each panel consists of three rows: top row, the low- and high-score group for the lncRNA signature in ccRCC patients; middle row, the survival status and duration of ccRCC cases; bottom row, heatmap showing the expression of the six key lncRNAs. The color, from blue to red shows, low to high expression, respectively

**Fig. 7:**
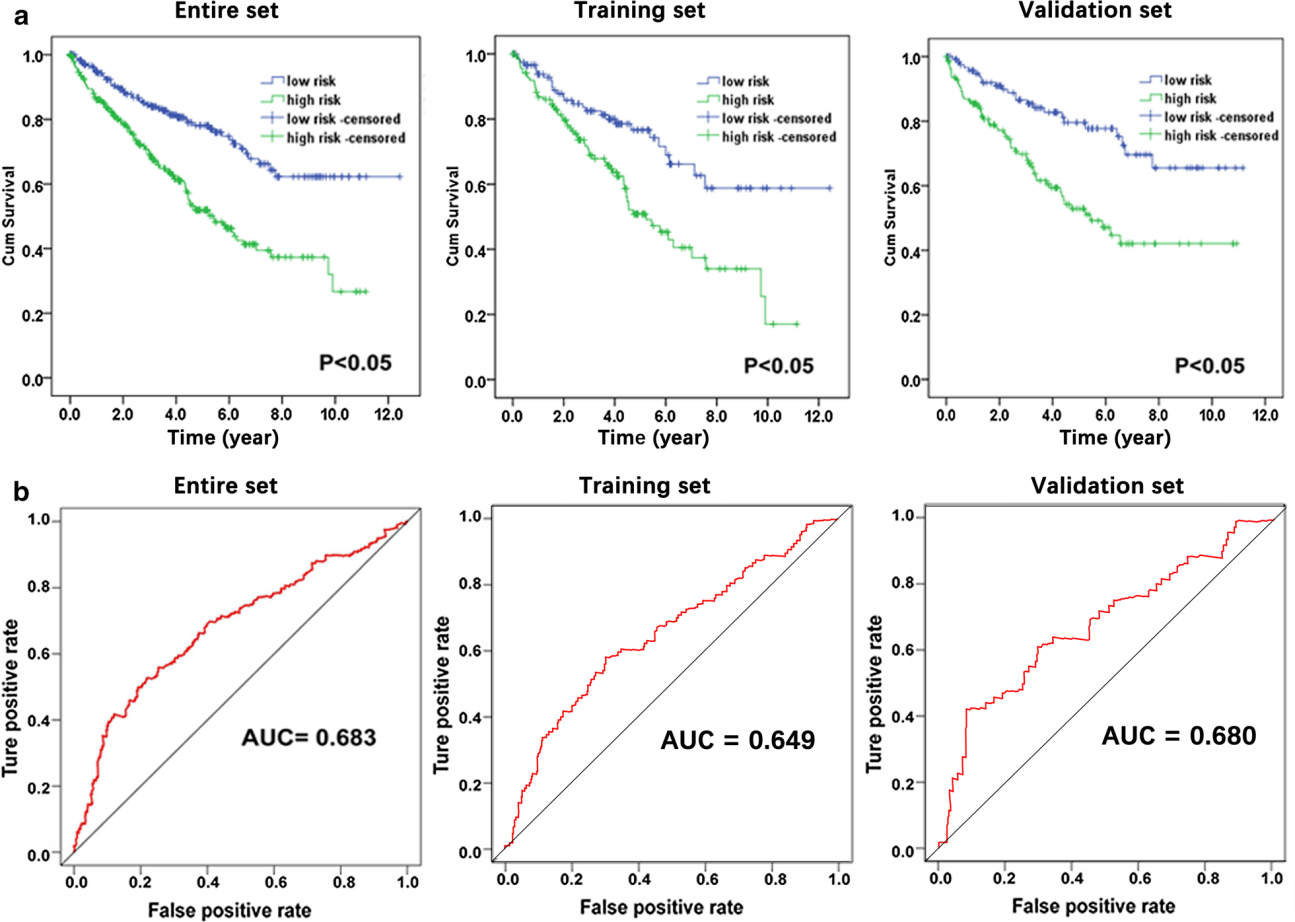
SurvivalROC curve and Kaplan–Meier curves for the six-lncRNA signature in the entire, training, and validation sets. **a** Kaplan–Meier survival curves showing overall survival outcomes for the high- and low-risk patients. **b** Time-dependent ROC curve analysis for survival prediction using the six-lncRNA signature

The ccRCC patients were classified into two cohorts, high- and low-risk groups, according to the cumulative distribution curve inflection point of the six-lncRNA-based risk score (Fig. 6). We gauged the differences in expression levels for these 6 lncRNAs between the high- and low-risk cohorts. Compared with the low-risk group, expression of LINC01510 was lower in the high-risk group, yet the expression of the other 5 lncRNAs was higher in the high-risk group (Fig. 6). K–M curves indicated that the median survival time of patients in the high-risk group was 73.5 months, which was much shorter than that of the low-risk group (112.6 months, *P* < 0.05; Fig. 7a). Furthermore, the risk score predicted 5-year survival of ccRCC patients across the entire set (AUC at 5 years, 0.683; C-index, 0.853; 95% CI 0.817–0.889). Moreover, the training and validation sets showed similar performance (AUC at 5 years, 0.649 and 0.680, respectively; C-index, 0.822 and 0.891; 95% CI 0.774–0.870 and 0.844–0.938) (Fig. 7). Additionally, the risk score HR generated by univariate Cox regression was 2.372 (95% CI 1.712–3.288, *P* < 0.001), and multivariate Cox proportional hazards regression analysis demonstrated an accordant HR of 1.693 (95% CI 1.181–2.425, *P* = 0.004), which confirmed that the six-lncRNA-based risk score was an independent indicator of ccRCC patient survival (Table 3).

**Table 3:** Univariate and multivariate Cox analyses for the prognostic value of clinical features in clear cell renal cell carcinoma (ccRCC) patients

Variables	Univariate				Multivariate			
	*P*	HR	LL	UL	*P*	HR	LL	UL
Risk score (high-risk/low-risk)	< 0.001	2.372	1.712	3.288	0.004	1.693	1.181	2.425
Race (Asian/black/white)	0.497	0.837	0.5	1.4	0.349	0.767	0.44	1.337
Gender (female/male)	0.76	1.049	0.77	1.431	0.498	1.126	0.799	1.588
Age (> 60/< 60)	< 0.001	1.739	1.283	2.356	0.004	1.624	1.17	2.252
T (T3–4/T1–2)	< 0.001	3.152	2.326	4.272	0.957	0.983	0.53	1.823
N (N1–NX/N0)	0.557	0.914	0.678	1.232	0.06	0.732	0.529	1.013
M (M1–MX/M0)	< 0.001	3.736	2.743	5.089	0.017	1.63	1.09	2.436
Cancer status (with tumor/tumor free)	< 0.001	5.008	3.611	6.946	< 0.001	3.182	2.176	4.653
Clinical stage (III–IV/I–II)	< 0.001	3.85	2.802	5.291	0.232	1.539	0.759	3.124
Grade (G3–4/G1–2)	< 0.001	2.668	1.893	3.759	0.029	1.531	1.045	2.243

**Fig. 8:**
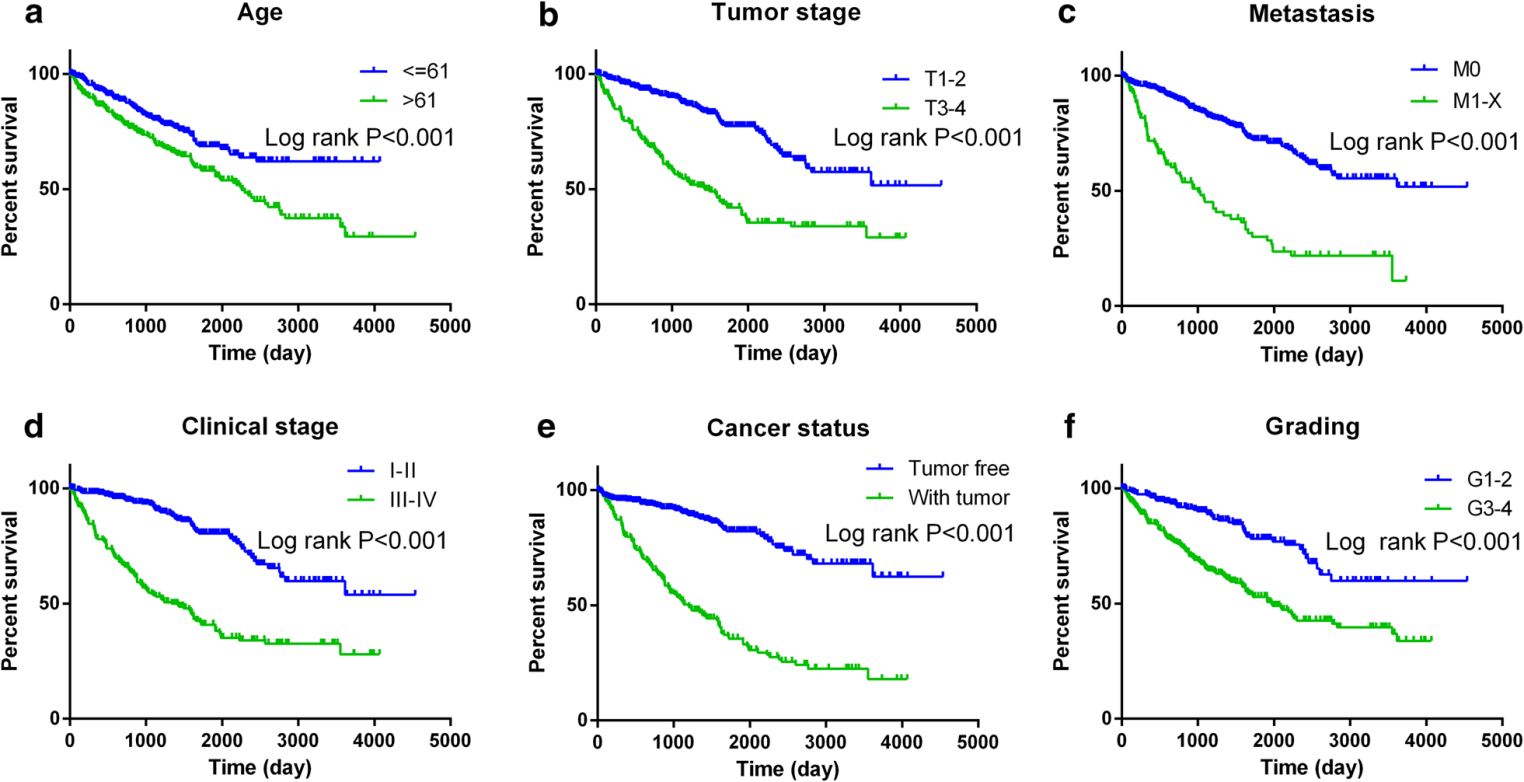
Kaplan–Meier survival curves in subgroup analyses according to different clinical factors. **a** Age (HR = 1.739, *P* < 0.01); **b** tumor stage (HR = 3.152, *P* < 0.001); **c** metastasis (HR = 3.736, *P* < 0.001); **d** cancer status (HR = 5.008, *P* < 0.001); **e** clinical stage (HR = 3.85, *P* < 0.001); **f** grade (HR = 2.668, *P* < 0.001)

Meanwhile, the prognostic value of a diversity of clinicopathological parameters was also explored. The K–M methodology revealed that the age, tumor stage, distant metastasis, cancer status, clinical stage, and grade could predict the outcome (Fig. 8). Some parameters were discovered to exhibit prognostic value through univariate analysis; nevertheless, it was demonstrated by multivariate analysis that age, metastasis, cancer status, and grade appeared statistically significant (Table 3).

**Fig. 9:**
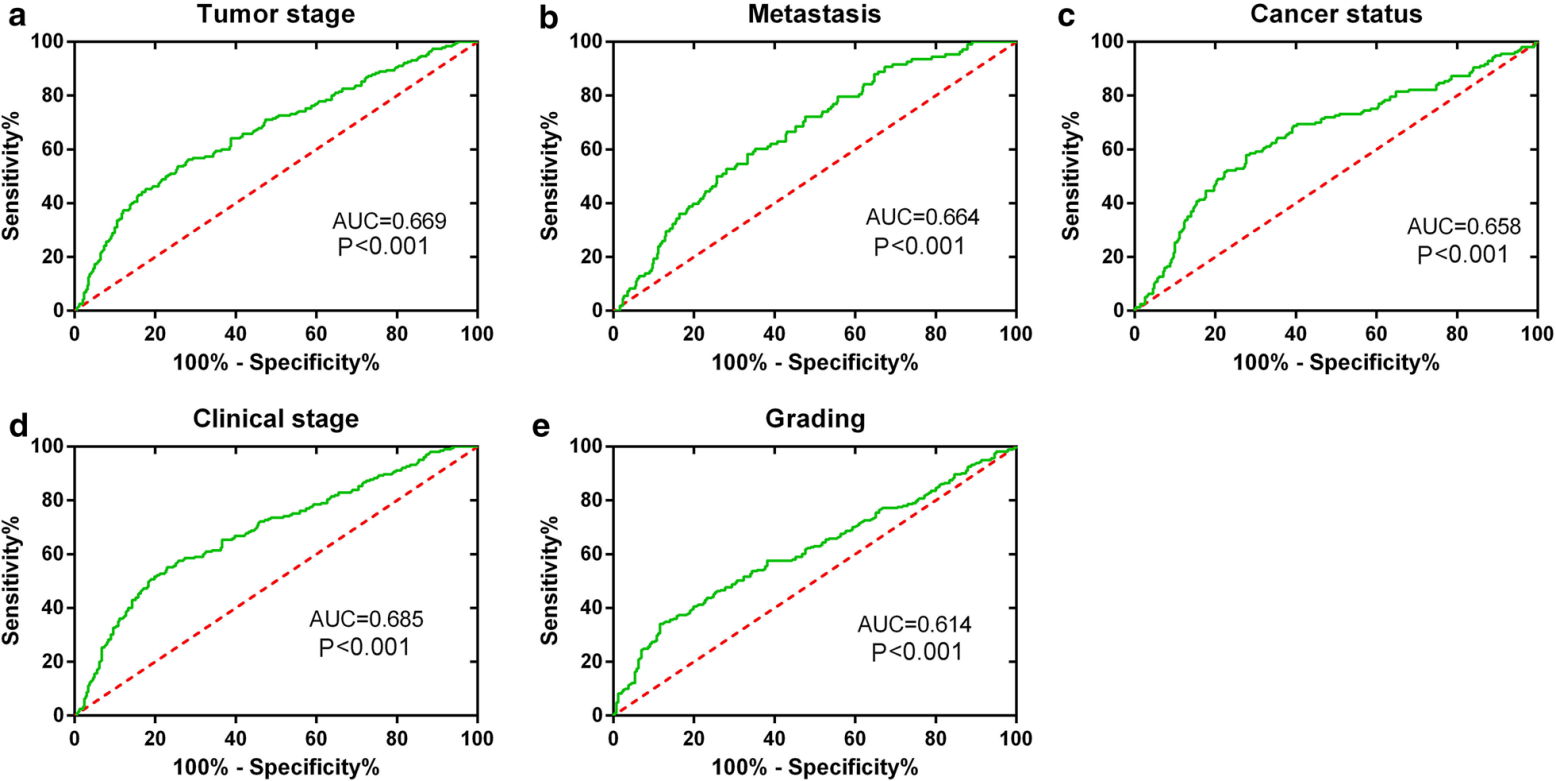
Predictive value of the risk scores for clinical features by receiver operating characteristic (ROC) curves. **a** Tumor stage (AUC = 0.669, *P* < 0.001); **b** distant metastasis (AUC = 0.664, *P *< 0.001); **c** cancer status (AUC = 0.658, *P* < 0.001); **d** advanced clinical stage (AUC = 0.685, *P* < 0.001), and **e** grade (AUC = 0.614, *P* < 0.001)

**Fig. 10:**
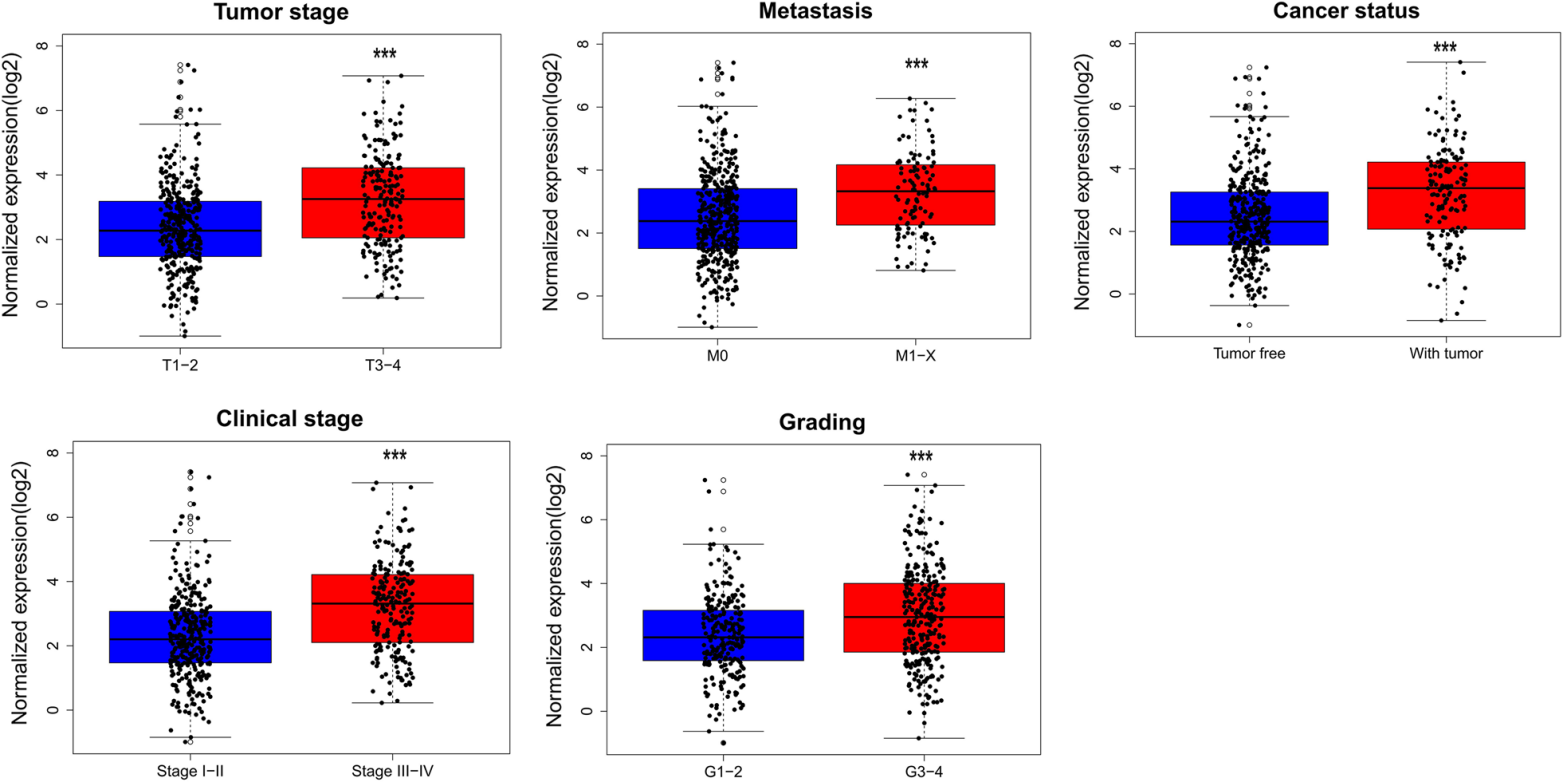
Association between the risk score and clinicopathological features in clear cell renal cell carcinoma (ccRCC). Statistically significant differences in risk score are noted for various clinicopathological features: tumor stage, metastasis, cancer status, clinical stage, and grade. *P < 0.05; **P < 0.01; ***P < 0.001

ROC analysis showed that the six-lncRNA-based risk score could significantly predict tumor progression, including tumor stage (AUC = 0.669, *P* < 0.001), distant metastasis (AUC = 0.664, *P* < 0.001), cancer status (AUC = 0.658, *P *< 0.001), advanced clinical stage (AUC = 0.685, *P* < 0.001), and grade (AUC = 0.614, *P* < 0.001). Additionally, associations between risk score and different clinical features were also found (Figs. 9 and 10 and Table 4).

**Table 4:** Association of the risk score of the six-lncRNA signature with clinical features in clear cell renal cell carcinoma (ccRCC) patients

Parameters	*N*	*t*-test				ROC				Spearman	
		Mean	SD	*t*	*P*	(AUC)	LL	UL	*P*	*r*	*P*
Age											
≤ 60	282	2.551701307	1.446528522	− 2.05	0.041	0.548	0.499	0.597	0.058	0.082	0.058
> 60	248	2.812258819	1.475766012								
Tumor stage											
T1–2	340	2.365372014	1.376057212	− 6.749	< 0.001	0.669	0.62	0.718	< 0.001	0.281	< 0.001
T3–T4	190	3.225228793	1.459955424								
Lympy node metastasis											
N0	239	2.692022362	1.38927271	0.262	0.794	0.481	0.432	0.531	0.462	− 0.032	0.462
N1–NX	291	2.658510691	1.526057572								
Metastasis											
M0	420	2.512539246	1.468891385	− 5.073	< 0.001	0.664	0.61	0.718	< 0.001	0.229	< 0.001
M1–MX	108	3.297433572	1.28906475								
Cancer status											
Tumor free	350	2.466519837	1.399223521	− 5.376	< 0.001	0.658	0.605	0.711	< 0.001	0.253	< 0.001
With tumor	157	3.207910769	1.514022946								
Clinical stage											
I–II	322	2.312078114	1.366475268	− 7.384	< 0.001	0.685	0.638	0.732	< 0.001	0.313	< 0.001
III–IV	205	3.226306599	1.415145295								
Grading											
G1–2	241	2.3761705	1.272204534	− 4.767	< 0.001	0.614	0.566	0.662	< 0.001	0.197	< 0.001
G3–4	281	2.967072624	1.559275235								

### Functional evaluation of the differentially expressed genes in high- and low-risk groups

**Fig. 11:**
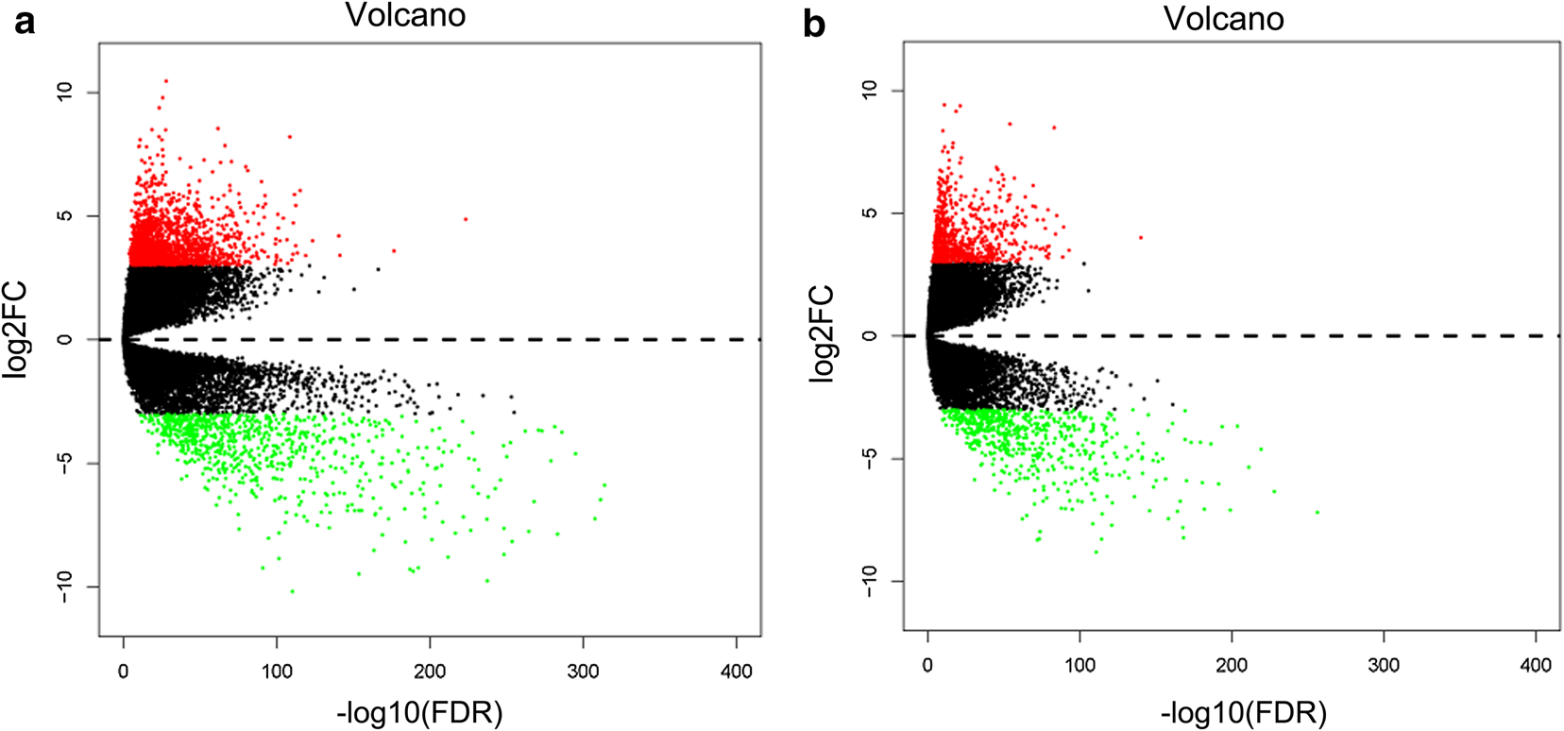
Volcano plots of differentially expressed genes (DEGs) in high- and low-risk groups. Volcano plots of DEGs were generated using the edgeR package in R with *P*_adj_ < 0.01 and |log_2_FC| > 3. **a** High-risk score group. **b** Low-risk score group

**Fig. 12:**
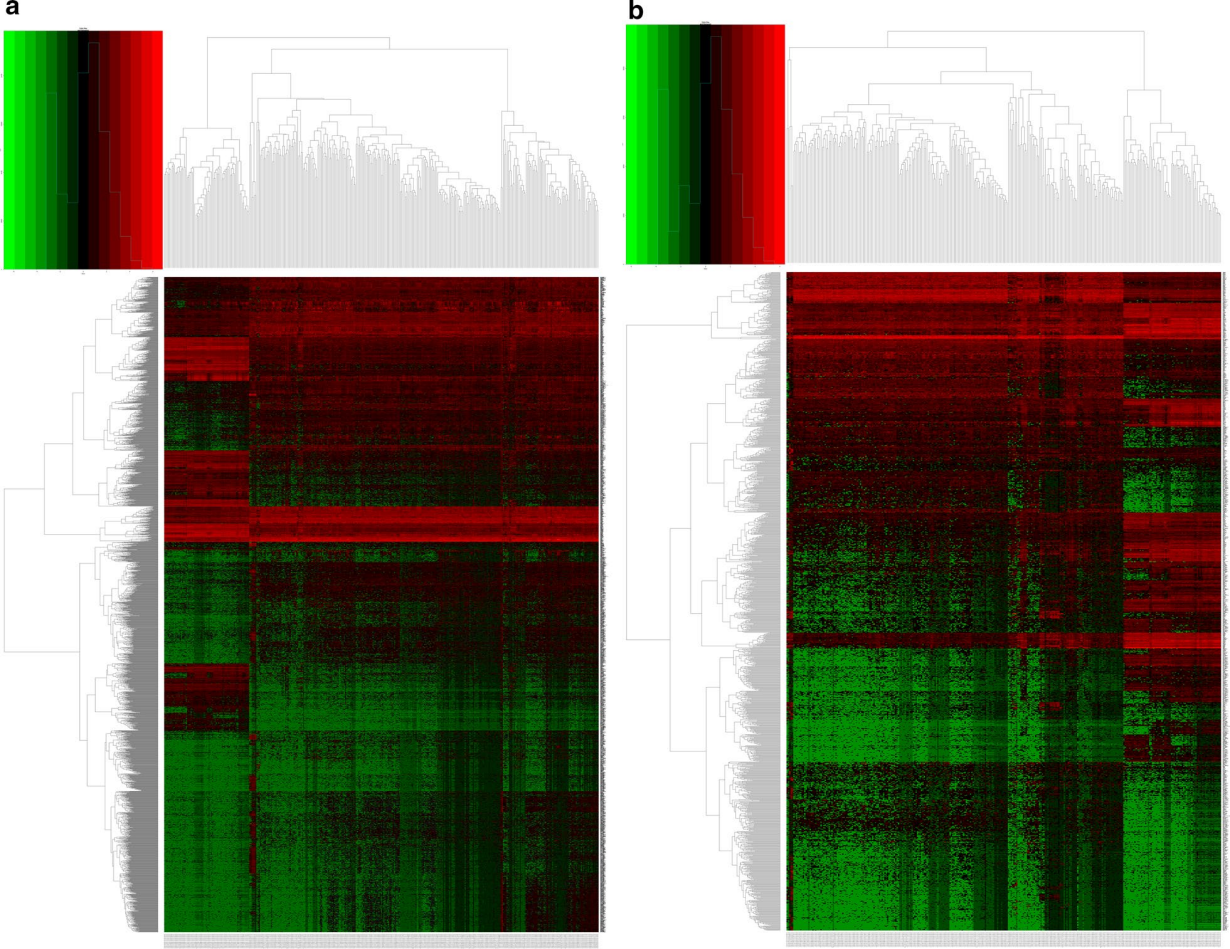
Heatmaps of differentially expressed genes (DEGs) in high- and low-risk groups. Heatmaps of DEGs were generated using the edgeR package in R with *P*_adj_ < 0.01 and |log_2_FC| > 3. **a** High-risk score group. **b** Low-risk score group

Volcano plots and heatmaps of DEGs in high/low-risk score group of ccRCC and normal kidney samples were created (Figs. 11 and 12). GO terms and KEGG pathways are shown in Additional file 2: Table S2, Additional file 3: Table S3, Additional file 4: Table S4, Additional file 5: Table S5 which suggests that different pathways were enriched between the high- and low-risk groups.

**Fig. 13:**
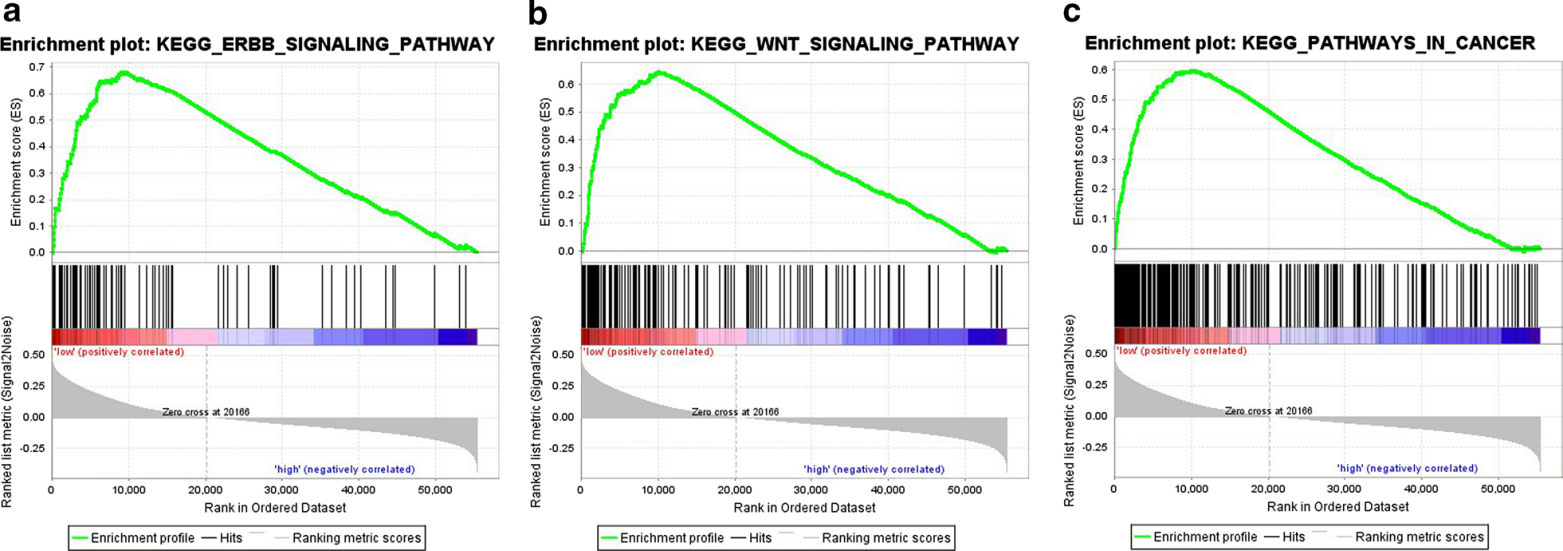
Gene set enrichment analysis (GSEA) identifies cancer-related KEGG pathways associated with risk score. GSEA validated the enhanced activity of **a** the ERBB signaling pathway, **b** WNT signaling pathway, and **c** pathway in cancers

GSEA was also performed to investigate related biological processes and signaling pathways [12]. We compared the gene profiles of ccRCC patients in the high- and low-risk groups categorized by the six-lncRNA-based risk score. The gene sets with significantly different expression (FDR < 0.25 and nominal *P* < 0.005) were used for GSEA. In total, 6 pathways were found to be significantly enriched in the high-risk group, including primary immunodeficiency, olfactory transduction, allograft rejection, autoimmune thyroid disease, and immune network for IgA production. By contrast, GSEA revealed that the gene sets in the low-risk group were enriched in 152 pathways including several cancer related pathways, such as the ERBB signaling pathway, WNT signaling, and the WNT pathway in cancer (Fig. 13). The associated biological pathways are shown in Tables 5 and 6 as assessed by GSEA, as well as in Additional file 2: Table S2, Additional file 3: Table S3, Additional file 4: Table S4, Additional file 5: Table S5. PPI networks were also analyzed for the genes involved in the ‘Renal cell carcinoma pathway,’ and several hub genes, such as PIK3CA, VEGFA, and PIK3CB were noted (Additional file 6: Fig. S1).

**Table 5:** Pathways enriched in the high-risk group according to gene set enrichment analysis (GSEA)

Name	Size	ES	NES	NOM *P*	FDR *q*	FWER *P*	Rank at max	Leading edge
KEGG_PRIMARY_IMMUNODEFICIENCY	35	0.486	1.787836	0	0.023534	0.121	9936	Tags = 49%, list = 18%, signal = 59%
KEGG_OLFACTORY_TRANSDUCTION	386	0.307	1.663547	0	0.040478	0.341	25,224	Tags = 57%, list = 46%, signal = 104%
KEGG_ALLOGRAFT_REJECTION	35	0.429	1.552546	0.019523	0.067792	0.668	9137	Tags = 31%, list = 16%, signal = 38%
KEGG_AUTOIMMUNE_THYROID_DISEASE	50	0.392	1.547779	0.00463	0.052901	0.685	27,174	Tags = 72%, list = 49%, signal = 141%
KEGG_GRAFT_VERSUS_HOST_DISEASE	37	0.419	1.5393	0.011876	0.045754	0.712	9137	Tags = 27%, list = 16%, signal = 32%
KEGG_INTESTINAL_IMMUNE_NETWORK_FOR_IGA_PRODUCTION	46	0.397	1.514021	0.01171	0.046609	0.784	12,802	Tags = 37%, list = 23%, signal = 48%
KEGG_TASTE_TRANSDUCTION	50	0.356	1.395092	0.065789	0.095099	0.973	12,317	Tags = 44%, list = 22%, signal = 57%
KEGG_ASTHMA	28	0.409	1.389118	0.073333	0.086951	0.976	8139	Tags = 25%, list = 15%, signal = 29%
KEGG_TYPE_I_DIABETES_MELLITUS	41	0.345	1.248997	0.139326	0.192182	1	9137	Tags = 29%, list = 16%, signal = 35%
KEGG_ALPHA_LINOLENIC_ACID_METABOLISM	19	0.405	1.245173	0.159751	0.177156	1	6773	Tags = 42%, list = 12%, signal = 48%
KEGG_LINOLEIC_ACID_METABOLISM	29	0.351	1.201192	0.193059	0.207408	1	6773	Tags = 31%, list = 12%, signal = 35%
KEGG_SYSTEMIC_LUPUS_ERYTHEMATOSUS	135	0.243	1.140861	0.184697	0.265071	1	22,435	Tags = 50%, list = 40%, signal = 84%
KEGG_CYTOKINE_CYTOKINE_RECEPTOR_INTERACTION	257	0.221	1.133512	0.114391	0.254958	1	11,262	Tags = 28%, list = 20%, signal = 35%
KEGG_HEMATOPOIETIC_CELL_LINEAGE	85	0.21	0.916435	0.667494	0.623275	1	8343	Tags = 20%, list = 15%, signal = 24%

**Table 6:** Pathways enriched in the low-risk group according to gene set enrichment analysis (GSEA)

Name	Size	ES	NES	NOM *P*	FDR *q*	FWER *P*	Rank at max	Leading edge
KEGG_UBIQUITIN_MEDIATED_PROTEOLYSIS	132	− 0.727497	− 3.170449	0	0	0	9024	Tags = 67%, list = 16%, signal = 79%
KEGG_OXIDATIVE_PHOSPHORYLATION	118	− 0.708458	− 3.066339	0	0	0	10,688	Tags = 70%, list = 19%, signal = 87%
KEGG_PEROXISOME	78	− 0.755321	− 3.048664	0	0	0	9185	Tags = 72%, list = 17%, signal = 86%
KEGG_ENDOCYTOSIS	176	− 0.666864	− 3.027353	0	0	0	8682	Tags = 59%, list = 16%, signal = 70%
KEGG_VALINE_LEUCINE_AND_ISOLEUCINE_DEGRADATION	44	− 0.818906	− 2.976677	0	0	0	5843	Tags = 80%, list = 11%, signal = 89%
KEGG_FOCAL_ADHESION	197	− 0.636304	− 2.969007	0	0	0	9112	Tags = 54%, list = 16%, signal = 65%
KEGG_PARKINSONS_DISEASE	114	− 0.687936	− 2.94244	0	0	0	8989	Tags = 61%, list = 16%, signal = 73%
KEGG_NEUROTROPHIN_SIGNALING_PATHWAY	125	− 0.679093	− 2.935972	0	0	0	9509	Tags = 62%, list = 17%, signal = 74%
KEGG_ALZHEIMERS_DISEASE	158	− 0.646806	− 2.935276	0	0	0	8989	Tags = 56%, list = 16%, signal = 67%
KEGG_HUNTINGTONS_DISEASE	174	− 0.64174	− 2.909377	0	0	0	8989	Tags = 56%, list = 16%, signal = 66%
KEGG_PROSTATE_CANCER	89	− 0.716898	− 2.907305	0	0	0	9112	Tags = 63%, list = 16%, signal = 75%
KEGG_LYSOSOME	119	− 0.674316	− 2.895725	0	0	0	9385	Tags = 64%, list = 17%, signal = 77%
KEGG_PATHWAYS_IN_CANCER	321	− 0.597361	− 2.89323	0	0	0	10,390	Tags = 53%, list = 19%, signal = 65%
KEGG_TIGHT_JUNCTION	128	− 0.653417	− 2.876733	0	0	0	9304	Tags = 61%, list = 17%, signal = 73%
KEGG_WNT_SIGNALING_PATHWAY	149	− 0.64552	− 2.851381	0	0	0	10,037	Tags = 56%, list = 18%, signal = 69%
KEGG_ADHERENS_JUNCTION	68	− 0.726372	− 2.841678	0	0	0	7905	Tags = 71%, list = 14%, signal = 82%
KEGG_ENDOMETRIAL_CANCER	52	− 0.750658	− 2.820001	0	0	0	9383	Tags = 75%, list = 17%, signal = 90%
KEGG_CHRONIC_MYELOID_LEUKEMIA	73	− 0.694979	− 2.816546	0	0	0	9112	Tags = 62%, list = 16%, signal = 74%
KEGG_REGULATION_OF_ACTIN_CYTOSKELETON	211	− 0.606974	− 2.81045	0	0	0	9156	Tags = 53%, list = 17%, signal = 63%
KEGG_CITRATE_CYCLE_TCA_CYCLE	30	− 0.842038	− 2.791856	0	0	0	5709	Tags = 83%, list = 10%, signal = 93%
KEGG_INSULIN_SIGNALING_PATHWAY	137	− 0.639309	− 2.781884	0	0	0	9112	Tags = 56%, list = 16%, signal = 67%
KEGG_ERBB_SIGNALING_PATHWAY	86	− 0.681992	− 2.769589	0	0	0	9112	Tags = 62%, list = 16%, signal = 74%

### Validation of these lncRNAs using Gene Expression Omnibus DataSets and International Cancer Genomics Consortium (ICGC) database

**Fig. 14:**
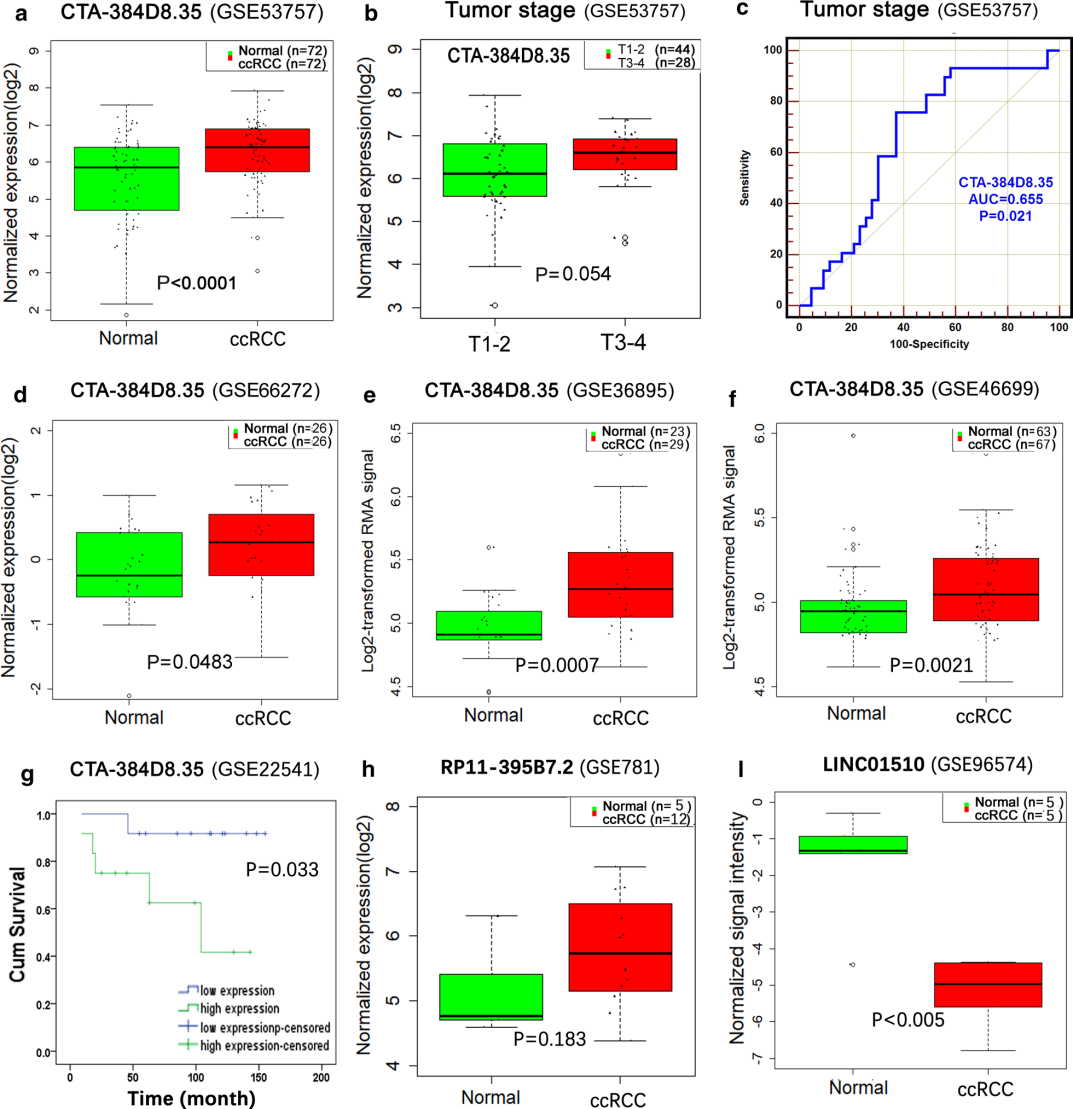
Validation of lncRNAs in clear cell renal cell carcinoma (ccRCC) based on Gene Expression Omnibus (GEO) data. **a** Boxplot showing expression of CTA-384D8.35 (GSE53757) in normal and ccRCC tissues. **b** The association of CTA-384D8.35 expression level with tumor (T) stage was also considered. **c** ROC curve of CTA-384D8.35 (GSE53757). **d** Boxplot showing expression of CTA-384D8.35 (GSE66272). **e** Boxplot showing expression of CTA-384D8.35 (GSE36895). **f** Boxplot showing expression of CTA-384D8.35 (GSE46699). **g** Kaplan–Meier survival curves of CTA-384D8.35 (GSE66272). **h** Boxplot showing expression of RP11-395B7.2 (GSE781). **i** Boxplot showing expression of LINC01510 (GSE96574)

In total, 4030 items (GSE = 248, GPL = 96) were identified from the GEO DataSets through our searching strategies. The standard process for retrieval and inclusion is shown in Additional file 7: Fig. S2. Some annotation for these 6 lncRNAs was found in the following platforms of GEO DataSets: GPL19615, GPL8841, GPL19197, GPL1707, GPL570, GPL5175, GPL15096, GPL97, and GPL96. Ultimately, only GPL19615 (GSE96574 contained LINC01510), GPL570 (GSE53757, GSE66272, GSE36895, GSE46699, and GSE22541 contained CTA-384D8.35) and GPL96 (GSE781 contained RP11-395B7.2) were included in subsequent analyses. The expression levels of CTA-384D8.35 and LINC01510 from these 6 microarrays were remarkably higher in ccRCC than those in normal controls (CTA-384D8.35: GSE53757 [*P* < 0.0001], GSE66272 [*P* = 0.0483], GSE36895 [*P* = 0.0007], GSE46699 [*P* = 0.0021]; LINC01510: GSE96574 [*P* < 0.005]), and the expression of RP11-395B7.2 also showed the same trend (*P* = 0.183). The AUC value of CTA-384D8.35 was 0.655 for anticipating advanced tumor stage, and CTA-384D8.35 had prognostic value for patients with ccRCC (*P* = 0.033). These results were consistent with our previous results based on TCGA data (Table 7, Fig. 14).

**Table 7:** Validation of lncRNA expression in clear cell renal cell carcinoma (ccRCC) based on Gene Expression Omnibus (GEO) data

Variable	GEO	NC			KIRC			t-test	
		n	mean	SD	n	mean	SD	t	P
CTA-384D8.35	GSE53757	72	5.524	0.1441	72	6.236	0.1001	4.054	< 0.0001
CTA-384D8.35	GSE66272	26	− 0.3785	0.2042	26	0.1507	0.1633	2.025	0.0483

*GEO* Gene Expression Omnibus, *NC* normal control, *ccRCC* clear cell renal cell carcinoma

**Fig. 15:**
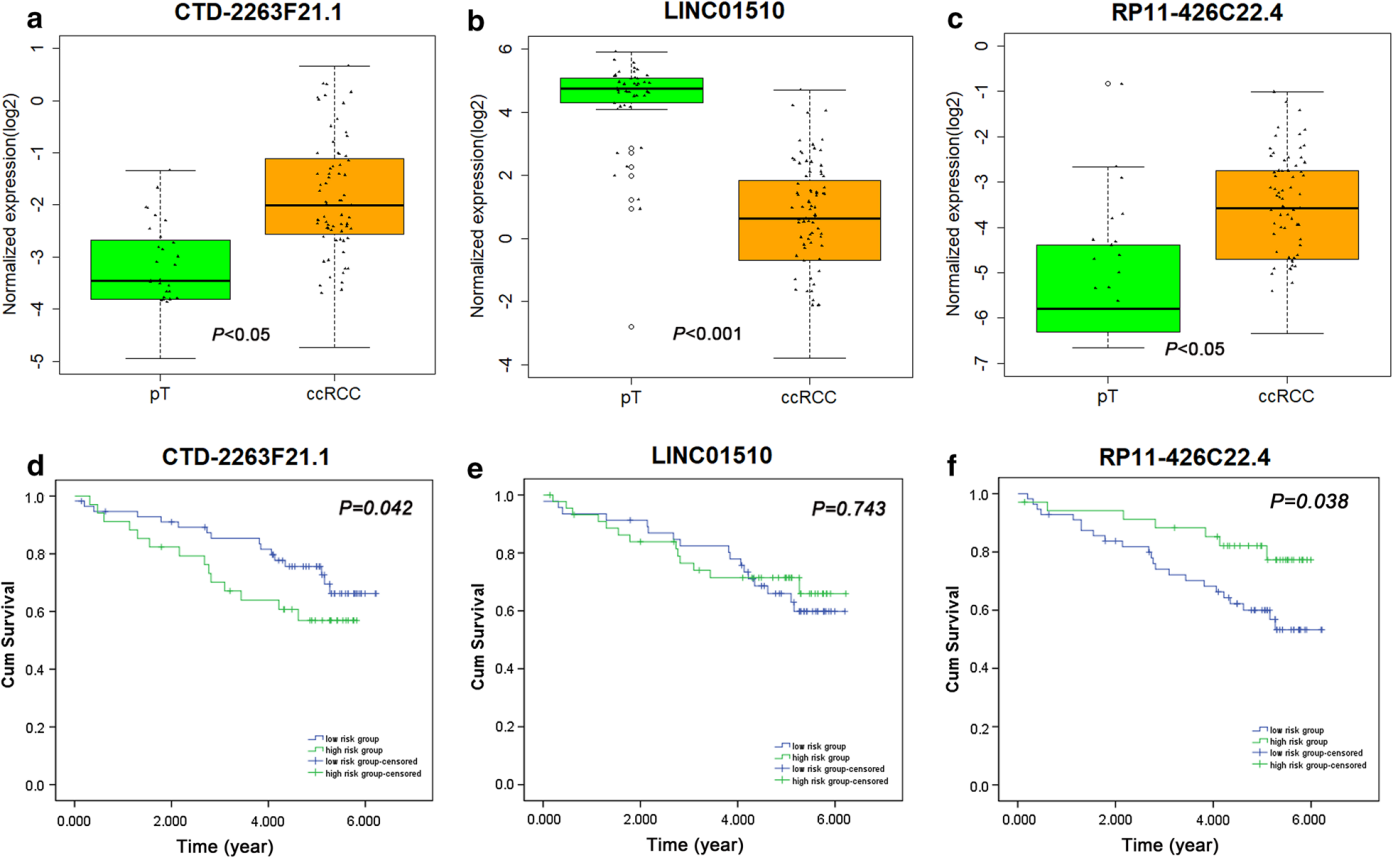
**a** Differential expression of CTD-2263F21.1 between clear cell renal cell carcinoma (ccRCC) and para-tumorous (pT) renal tissues (*P* < 0.05). **b** Differential expression of LINC01510 between clear cell renal cell carcinoma (ccRCC) and para-tumorous (pT) renal tissues (*P* < 0.001). **c** Differential expression of RP11-426C22.4 between clear cell renal cell carcinoma (ccRCC) and para-tumorous (pT) renal tissues (*P* < 0.05). **d** Kaplan–Meier survival curve of CTD-2263F21.1 (*P* = 0.042). **e** Kaplan–Meier survival curve of LINC01510 (*P* = 0.743). **f** Kaplan–Meier survival curve of RP11-426C22.4 (*P* = 0.038)

Renal Cell Cancer (RECA-EU) data was selected from the International Cancer Genomics Consortium (ICGC) database, containing 91 ccRCC tissues and 45 adjacent non-tumorous renal tissue samples. Three of the six lncRNAs were matched, including CTD-2263F21.1, LINC01510 and RP11-426C22.4. Differential expression and prognostic value analysis of these three lncRNAs were performed. The differential expression of these three lncRNAs was meaningful (*P *< 0.05) and consistent with the results of TCGA. Kaplan–Meier survival curves of CTD-2263F21.1 and RP11-426C22.4 also showed the value of their predicted survival (*P *< 0.05) (Fig. 15).

## Discussion

This study analyzed TCGA sequencing data to discover effective prognostic biomarkers for ccRCC, which have the potential to guide future clinical and basic medical studies. First, we analyzed the statistical significance of differentially-expressed lncRNAs in ccRCC patients using the R packages edgeR and DESeq, and systematically assessed their prognostic value. Notably, the best prognostic value was achieved using a pool that consisted of 6 lncRNAs (CTA-384D8.35, CTD-2263F21.1, LINC01510, RP11-352G9.1, RP11-395B7.2, and RP11-426C22.4), which were obtained via multivariate Cox regression. The resulting six-lncRNA-based risk score accurately predicted the progression and prognosis of ccRCC. With ccRCC patients classified into high- and low-risk groups, we discovered that differentially-expressed genes in these two groups were dissimilar, and the essential signaling pathways were unique as well (Additional file 8: Fig. S3).

Some ccRCC studies have already utilized lncRNA expression profiling. Similarly, studies on lncRNA interactions with other molecules have been on the rise in recent years. The most frequently used research techniques for assessing lncRNA expression profiles of renal cell carcinoma (RCC) include microarray assays and ChIP-Seq experiments [16, 35–38]. However, these studies were limited by their small sample sizes and insufficient focal lncRNAs. In 2018, Liu et al. published a paper on a novel lncRNA profile reveals potential prognostic biomarkers in clear cell renal cell carcinoma. The expression profile of 1801 lncRNAs of ccRCC patients was obtained using TCGA RNASeqv2 system [39]. To enable a comprehensive understanding of lncRNAs in ccRCC, the present study mined high-throughput TCGA data from 530 patients and analyzed 13,198 lncRNAs. 869 differentially expressed lncRNAs were assessed using edgeR and DESeq packages, and used for subsequent analysis. In the study of Qu et al. and Liu et al., there were only 51 and 247 differentially expressed lncRNAs, respectively [39, 40].

Several studies have revealed that abnormal expression levels of lncRNAs are correlated with OS, 5-year survival, disease-free survival, disease grade and stage, recurrence, and metastasis. However, each previous study mainly focused on a single lncRNA. For example, an undesirable prognosis for RCC patients was connected with decreased expression of the lncRNAs NONHSAT123350, CADM1-AS1, TCL6, and lnc-ZNF180-2 [41]. Furthermore, increased expression of SPRY4-IT1, RCCRT1, MALAT1, LINC00152, and PVT1 also indicated unsatisfactory results [41]. Owing to the popularity of high-throughput TCGA data, the use of sequencing data was considered an ideal approach to discover novel lncRNAs. Therefore, using multiple statistical methods for prognostic analysis, we found that CTA-384D8.35, CTD-2263F21.1, LINC01510, RP11-352G9.1, RP11-395B7.2, and RP11-426C22.4 were of great prognostic value. More importantly, the pool composed of these 6 lncRNAs was the basis for a risk score that provided a superior means of predicting disease progression and prognosis.

Very recently, Shi et al. [42] used TCGA reads per kilobase of exon model per million mapped reads (RPKM) data to categorize 9669 lncRNAs from 440 kidney cancer patients into a training set (n = 220) and a testing set (n = 220). They discovered that expression of a five-lncRNA signature (consisting of AC069513.4, AC003092.1, CTC-205M6.2, RP11-507K2.3, and U91328.21) was closely associated with kidney cancer patient OS. Using the training set, lncRNAs were identified with a univariate Cox regression model, and these five lncRNAs were closely linked to patient OS. The five-lncRNA-based risk score was confirmed in both the testing set and the entire set. However, the results of Shi et al. [42] were inconsistent with ours as the five lncRNAs in their study did not overlap with the six lncRNAs in ours. However, the analysis that we conducted had the following advantages. First, more samples were included in our study (*n* = 539). Second, more lncRNAs were annotated (*n* = 13,198). Third, we simply analyzed those differentially-expressed lncRNAs for their prognostic value. If lncRNAs exerted inconsiderable influences on tumorigenesis, their prognostic value would be diminished. Two of the five lncRNAs reported in the study by Shi et al. [42] (U91328.21 and CTC-205M6.2) showed no remarkable differences in expression between ccRCC and non-cancerous renal tissues (Additional file 9: Fig. S4). The reason for this result may be that the value of RPKM data was not suitable for using edgeR to analyze differentially expressed genes [43]. We investigated the prognostic significance of the six lncRNAs identified in the present study based on the premise that their expression patterns exhibited noticeable differences between cancerous and non-cancerous tissues. Consequently, the six lncRNAs identified in the present study (i.e., CTA-384D8.35, CTD-2263F21.1, LINC01510, RP11-352G9.1, RP11-395B7.2, and RP11-426C22.4) functioned not only at the outset of tumorigenesis but also in tumor progression. Fourth, taking other factors into consideration, we applied multivariate Cox proportional hazards regression analysis to discover novel biomarkers with prognostic value, which guaranteed a more valid and comprehensive result. Fifth, the ccRCC dataset was divided into training and validation sets to verify the prognostic efficacy of the six-lncRNA-based signature. Sixth, using GEO and ICGC datasets for validation, we found a ccRCC-related series consisting of 248 samples from the GEO Datasets. CTA-384D8.35, CTD-2263F21.1 and RP11-426C22.4 had prognostic value for patients with ccRCC, and the clinical value of three lncRNAs (CTA-384D8.35, RP11-395B7.2, and LINC01510) was also partly verified by six microarrays. Lastly, a total of 530 cases of ccRCC were divided into high- and low-risk groups, and differences in pathways between the two groups were also investigated. Moreover, the potential signaling pathways and molecular mechanism in ccRCC were explored for their influences on prognosis.

Through GSEA, it was determined that the six novel lncRNAs may play unique roles in ccRCC via specific signaling pathways. ‘Pathway in cancer’ (321 genes) includes multiple pathways, such as the ‘Renal cell carcinoma pathway’ (49 genes). Hub genes in the ‘Renal cell carcinoma pathway’ based on PPI analysis, such as PIK3CA, VEGFA, and PIK3CB, were noted and have also been observed to play vital roles in ccRCC [44–49]. Interestingly, PIK3CA has been identified as a direct target of miR-490-5p and miR-19a in renal carcinoma [44, 45]. VEGFA was the most important trigger for angiogenesis [46], and it was the target of miR-185, which acted as a tumor suppressor in ccRCC [47]. VEGFA was also reported to act as a stimulus of ccRCC cell migration, invasion, and angiogenesis [48]. Thus, these six novel lncRNAs may begin their function by activating genes in the ‘Renal cell carcinoma pathway.’ In addition to the ‘Renal cell carcinoma pathway,’ by modulating the ‘Wnt signaling pathway,’ the lncRNAs CCAT2 and Kindlin‑2 appear to promote clear cell renal cell carcinoma progression [50, 51]. We also found that the top three KEGG pathways for DEGs of patients in the high-risk group included KEGG_PRIMARY_IMMUNODEFICIENCY, KEGG_OLFACTORY_TRANSDUCTION, and KEGG_ALLOGRAFT_REJECTION, while in the low-risk group the three most dominant pathways were KEGG_UBIQUITIN_MEDIATED_PROTEOLYSIS, KEGG_OXIDATIVE_PHOSPHORYLATION, and KEGG_PEROXISOME. There were some identified pathways that differed between the high- and the low-risk groups. As the six lncRNAs that we detected were novel and no relevant research has been conducted on their functions, the above analysis of the signaling pathways offers prospects into future research on their molecular mechanisms.

In many cancers, gene expression signatures and prognostic models have proven to be useful tools for predicting clinical outcomes and prognostic value based on molecular characteristics that drive pathogenesis. For example, Brooks et al. [52] developed a 34-gene subtype predictor to classify ccRCC tumors according to good risk (ccA) and poor risk (ccB) subtypes and built a subtype-inclusive model to predict patient survival outcomes. Their model provides prognostic stratification and improves the established algorithms to assess risk of recurrence and death in patients with non-metastatic ccRCC. However, the detection of 34 indicators presents a significant clinical burden. Additionally, a 16-gene recurrence score (RS) assay was developed and validated previously to predict the risk of disease recurrence in patients with stage I–III RCC after nephrectomy [53]. This study used data from the phase-III adjuvant sunitinib (S-TRAC) trial in high-risk phase-III RCC to provide additional validation of the 16-gene RS assay. The strong prognostic performance of the 16-gene RS assay was confirmed in the S-TRAC study, and the RS assay is now supported by IB level data. However, primary analysis focused on patients with T3 RCC and additional studies are needed to determine if RS predicts adjuvant treatment benefits. The (cell cycle progression) CCP score, based on levels of 31 cell cycle genes and 15 control genes from the tumor, had prognostic value in predicting metastatic progression after resection of organ-confined ccRCC by univariate analysis and multivariate logistic regression modeling [54]. The CCP score also had prognostic utility in a second TCGA renal cancer cohort with M1 metastasis at time of surgery. However, because the study cohort was relatively small, other genes in addition to CCP genes may still provide meaningful prognostic information. Because the assay used here was originally derived from prostate cancer, the ideal ccRCC gene set may differ from the genes evaluated in this study.

## Conclusion

In conclusion, by using TCGA data to evaluate lncRNAs from 530 ccRCC patients, we developed an effective six-lncRNA-based risk score, which has potential as a novel prognostic biomarker for ccRCC. However, this clinical finding needs further confirmation. Additionally, the function and molecular mechanisms of these novel lncRNAs also require in vitro and in vivo exploration.

## Supplementary information

**Additional file 1: Table S1.** Biological annotation of six prognostic lncRNAs.

**Additional file 2: Table S2.** Gene Ontology (GO) analysis in the high-risk score group.

**Additional file 3: Table S3.** Gene Ontology (GO) analysis in the low-risk score group.

**Additional file 4: Table S4.** Kyoto Encyclopedia of Genes and Genomes (KEGG) pathway analysis in the high-risk score group.

**Additional file 5: Table S5.** Kyoto Encyclopedia of Genes and Genomes (KEGG) pathway analysis in the low-risk score group.

**Additional file 6: Fig. S1.** Protein–protein interaction (PPI) network of genes from the ‘Renal cell carcinoma pathway.

**Additional file 7: Fig. S2.** Flowchart for lncRNA validation in clear cell renal cell carcinoma (ccRCC) based on Gene Expression Omnibus (GEO) data.

**Additional file 8: Fig. S3.** Flow chart summarizing the current study.

**Additional file 9: Fig. S4.** Differential expression of the five identified lncRNAs between clear cell renal cell carcinoma (ccRCC) and para-tumorous renal tissues in the study by Shi et al.

## Data Availability

The datasets used and/or analyzed during the current study are available from the corresponding author on reasonable request.

## Abbreviations

ccRCC: clear cell renal cell carcinoma
lncRNAs: long non-coding RNAs
TCGA: The Cancer Genome Atlas
RNA-Seq: RNA sequencing
OS: overall survival
ROC: receiver operating characteristic
K–M: Kaplan–Meier
C-index: concordance index
GSEA: gene set enrichment analysis
FDR: false discovery rate
PPI: protein–protein interaction
STRING: the Search Tool for the Retrieval of Interacting Genes
DEGs: differentially expressed genes
GO: Gene Ontology
KEGG: Kyoto Encyclopedia of Genes and Genomes
GEO: Gene Expression Omnibus
ICGC: International Cancer Genomics Consortium
RCC: renal cell carcinoma
RS: recurrence score
CCP: cell cycle progression
RPKM: reads per kilobase of exon model per million mapped reads

## References

[1] Lasser C, Shelke GV, Yeri A, Kim DK, Crescitelli R, Raimondo S, Sjostrand M, Gho YS, Van Keuren Jensen K, Lotvall J (2017). Two distinct extracellular RNA signatures released by a single cell type identified by microarray and next-generation sequencing. RNA Biol.

[2] Baras AS, Mitchell CJ, Myers JR, Gupta S, Weng LC, Ashton JM, Cornish TC, Pandey A, Halushka MK (2015). miRge—a multiplexed method of processing small RNA-Seq data to determine microRNA entropy. PLoS ONE.

[3] Ravo M, Cordella A, Rinaldi A, Bruno G, Alexandrova E, Saggese P, Nassa G, Giurato G, Tarallo R, Marchese G, Rizzo F, Stellato C, Biancardi R, Troisi J, Di Spiezio Sardo A, Zullo F, Weisz A, Guida M (2015). Small non-coding RNA deregulation in endometrial carcinogenesis. Oncotarget.

[4] Hombach S, Kretz M (2016). Non-coding RNAs: classification, biology and functioning. Adv Exp Med Biol.

[5] Wei Z, Batagov AO, Carter DR, Krichevsky AM (2016). Fetal bovine serum RNA interferes with the cell culture derived extracellular RNA. Sci Rep.

[6] Lefebvre FA, Benoit Bouvrette LP, Perras L, Blanchet-Cohen A, Garnier D, Rak J, Lecuyer E (2016). Comparative transcriptomic analysis of human and *Drosophila* extracellular vesicles. Sci Rep.

[7] Siegel RL, Miller KD, Jemal A (2017). Cancer statistics, 2017. CA Cancer J Clin.

[8] Chen W, Zheng R, Baade PD, Zhang S, Zeng H, Bray F, Jemal A, Yu XQ, He J (2016). Cancer statistics in China, 2015. CA Cancer J Clin.

[9] Kuthi L, Jenei A, Hajdu A, Nemeth I, Varga Z, Bajory Z, Pajor L, Ivanyi B (2016). Prognostic factors for renal cell carcinoma subtypes diagnosed according to the 2016 WHO renal tumor classification: a study involving 928 patients. Pathol Oncol Res.

[10] Malouf GG, Su X, Zhang J, Creighton CJ, Ho TH, Lu Y, Raynal NJ, Karam JA, Tamboli P, Allanick F, Mouawad R, Spano JP, Khayat D, Wood CG, Jelinek J, Tannir NM (2016). DNA methylation signature reveals cell ontogeny of renal cell carcinomas. Clin Cancer Res.

[11] Zhao H, Leppert JT, Peehl DM (2016). A protective role for androgen receptor in clear cell renal cell carcinoma based on mining TCGA data. PLoS ONE.

[12] He X, Sun F, Guo F, Wang K, Gao Y, Feng Y, Song B, Li W, Li Y (2017). Knockdown of long noncoding RNA FTX inhibits proliferation, migration, and invasion in renal cell carcinoma cells. Oncol Res.

[13] Chen J, Chen Y, Gu L, Li X, Gao Y, Lyu X, Chen L, Luo G, Wang L, Xie Y, Duan J, Peng C, Ma X (2016). LncRNAs act as prognostic and diagnostic biomarkers in renal cell carcinoma: a systematic review and meta-analysis. Oncotarget.

[14] Cao Y, Xu R, Xu X, Zhou Y, Cui L, He X (2016). Downregulation of lncRNA CASC2 by microRNA-21 increases the proliferation and migration of renal cell carcinoma cells. Mol Med Rep.

[15] Liu Z, Yan HY, Xia SY, Zhang C, Xiu YC (2016). Downregulation of long non-coding RNA TRIM52-AS1 functions as a tumor suppressor in renal cell carcinoma. Mol Med Rep.

[16] Liu H, Chen P, Jiang C, Han J, Zhao B, Ma Y, Mardan M (2016). Screening for the key lncRNA targets associated with metastasis of renal clear cell carcinoma. Medicine.

[17] Xiao H, Tang K, Liu P, Chen K, Hu J, Zeng J, Xiao W, Yu G, Yao W, Zhou H, Li H, Pan Y, Li A, Ye Z, Wang J, Xu H, Huang Q (2015). LncRNA MALAT1 functions as a competing endogenous RNA to regulate ZEB2 expression by sponging miR-200s in clear cell kidney carcinoma. Oncotarget.

[18] Gierlinski M, Cole C, Schofield P, Schurch NJ, Sherstnev A, Singh V, Wrobel N, Gharbi K, Simpson G, Owen-Hughes T, Blaxter M, Barton GJ (2015). Statistical models for RNA-seq data derived from a two-condition 48-replicate experiment. Bioinformatics.

[19] Tang W, Liao Z, Zou Q (2016). Which statistical significance test best detects oncomiRNAs in cancer tissues? An exploratory analysis. Oncotarget.

[20] Tang RX, Chen WJ, He RQ, Zeng JH, Liang L, Li SK, Ma J, Luo DZ, Chen G (2017). Identification of a RNA-Seq based prognostic signature with five lncRNAs for lung squamous cell carcinoma. Oncotarget.

[21] Zeng JH, Liang L, He RQ, Tang RX, Cai XY, Chen JQ, Luo DZ, Chen G (2017). Comprehensive investigation of a novel differentially expressed lncRNA expression profile signature to assess the survival of patients with colorectal adenocarcinoma. Oncotarget.

[22] Xu W, Xu B, Yao Y, Yu X, Cao H, Zhang J, Liu J, Sheng H (2016). Overexpression and biological function of IQGAP3 in human pancreatic cancer. Am J Transl Res.

[23] Langkilde A, Olsen LC, Saetrom P, Drablos F, Besenbacher S, Raaby L, Johansen C, Iversen L (2016). Pathway analysis of skin from psoriasis patients after adalimumab treatment reveals new early events in the anti-inflammatory mechanism of anti-TNF-alpha. PLoS ONE.

[24] Liu P, Sun M, Jiang W, Zhao J, Liang C, Zhang H (2016). Identification of targets of miRNA-221 and miRNA-222 in fulvestrant-resistant breast cancer. Oncol Lett.

[25] Sumimoto H, Takano A, Teramoto K, Daigo Y (2016). RAS-mitogen-activated protein kinase signal is required for enhanced PD-L1 expression in human lung cancers. PLoS ONE.

[26] Ma B, Liao T, Wen D, Dong C, Zhou L, Yang S, Wang Y, Ji Q (2016). Long intergenic non-coding RNA 271 is predictive of a poorer prognosis of papillary thyroid cancer. Sci Rep.

[27] Wang XL, Shi WP, Shi HC, Lu SC, Wang K, Sun C, He JS, Jin WG, Lv XX, Zou H, Shu YS (2016). Knockdown of TRIM65 inhibits lung cancer cell proliferation, migration and invasion: a therapeutic target in human lung cancer. Oncotarget.

[28] Zeng JH, Xiong DD, Pang YY, Zhang Y, Tang RX, Luo DZ, Chen G (2017). Identification of molecular targets for esophageal carcinoma diagnosis using miRNA-seq and RNA-seq data from The Cancer Genome Atlas: a study of 187 cases. Oncotarget.

[29] Xia L, Li D, Lin C, Ou S, Li X, Pan S (2017). Comparative study of joint bioinformatics analysis of underlying potential of ‘neurimmiR’, miR-212-3P/miR-132-3P, being involved in epilepsy and its emerging role in human cancer. Oncotarget.

[30] Ilias A, Lagnel J, Kapantaidaki DE, Roditakis E, Tsigenopoulos CS, Vontas J, Tsagkarakou A (2015). Transcription analysis of neonicotinoid resistance in Mediterranean (MED) populations of *B. tabaci* reveal novel cytochrome P450s, but no nAChR mutations associated with the phenotype. BMC Genomics.

[31] Zhao J, Zeng X, Song P, Wu X, Shi H (2016). AKT1 as the PageRank hub gene is associated with melanoma and its functional annotation is highly related to the estrogen signaling pathway that may regulate the growth of melanoma. Oncol Rep.

[32] Ambatipudi S, Gerstung M, Pandey M, Samant T, Patil A, Kane S, Desai RS, Schaffer AA, Beerenwinkel N, Mahimkar MB (2012). Genome-wide expression and copy number analysis identifies driver genes in gingivobuccal cancers. Genes Chromosomes Cancer.

[33] Cui X, Li Y, Yang L, You L, Wang X, Shi C, Ji C, Guo X (2016). Peptidome analysis of human milk from women delivering macrosomic fetuses reveals multiple means of protection for infants. Oncotarget.

[34] Yang L, Lin XL, Liang W, Fu SW, Lin WF, Tian XQ, Gao YJ, Chen HY, Dai J, Ge ZZ (2017). High expression of GPR116 indicates poor survival outcome and promotes tumor progression in colorectal carcinoma. Oncotarget.

[35] Deng M, Blondeau JJ, Schmidt D, Perner S, Muller SC, Ellinger J (2015). Identification of novel differentially expressed lncRNA and mRNA transcripts in clear cell renal cell carcinoma by expression profiling. Genomics Data.

[36] Blondeau JJ, Deng M, Syring I, Schrodter S, Schmidt D, Perner S, Muller SC, Ellinger J (2015). Identification of novel long non-coding RNAs in clear cell renal cell carcinoma. Clin Epigenet.

[37] Fachel AA, Tahira AC, Vilella-Arias SA, Maracaja-Coutinho V, Gimba ER, Vignal GM, Campos FS, Reis EM, Verjovski-Almeida S (2013). Expression analysis and in silico characterization of intronic long noncoding RNAs in renal cell carcinoma: emerging functional associations. Mol Cancer.

[38] He HT, Xu M, Kuang Y, Han XY, Wang MQ, Yang Q (2016). Biomarker and competing endogenous RNA potential of tumor-specific long noncoding RNA in chromophobe renal cell carcinoma. Oncotargets Ther.

[39] Liu T, Sui J, Zhang Y, Zhang XM, Wu WJ, Yang S, Xu SY, Hong WW, Peng H, Yin LH, Pu YP, Liang GY (2018). Comprehensive analysis of a novel lncRNA profile reveals potential prognostic biomarkers in clear cell renal cell carcinoma. Oncol Rep.

[40] Qu L, Wang ZL, Chen Q, Li YM, He HW, Hsieh JJ, Xue S, Wu ZJ, Liu B, Tang H, Xu XF, Xu F, Wang J, Bao Y, Wang AB, Wang D, Yi XM, Zhou ZK, Shi CJ, Zhong K, Sheng ZC, Zhou YL, Jiang J, Chu XY, He J, Ge JP, Zhang ZY, Zhou WQ, Chen C, Yang JH, Sun YH, Wang LH (2018). Prognostic value of a long non-coding rna signature in localized clear cell renal cell carcinoma. Eur Urol..

[41] Li M, Wang Y, Cheng L, Niu W, Zhao G, Raju JK, Huo J, Wu B, Yin B, Song Y, Bu R (2017). Long non-coding RNAs in renal cell carcinoma: a systematic review and clinical implications. Oncotarget.

[42] Shi D, Qu Q, Chang Q, Wang Y, Gui Y, Dong D (2017). A five-long non-coding RNA signature to improve prognosis prediction of clear cell renal cell carcinoma. Oncotarget.

[43] Chen K, Zeng J, Tang K, Xiao H, Hu J, Huang C, Yao W, Yu G, Xiao W, Guan W, Guo X, Xu H, Ye Z (2016). miR-490-5p suppresses tumour growth in renal cell carcinoma through targeting PIK3CA. Biol Cell.

[44] Varet H, Brillet-Guéguen L, Coppée JY, Dillies MA (2016). SARTools: a DESeq2- and EdgeR-based R pipeline for comprehensive differential analysis of RNA-Seq data. PLoS ONE.

[45] Xiao W, Gao Z, Duan Y, Yuan W, Ke Y (2015). Downregulation of miR-19a exhibits inhibitory effects on metastatic renal cell carcinoma by targeting PIK3CA and inactivating Notch signaling in vitro. Oncol Rep.

[46] Ebos JM (2015). Prodding the beast. Assessing the impact of treatment-induced metastasis. Cancer Res.

[47] Ma X, Shen D, Li H, Zhang Y, Lv X, Huang Q, Gao Y, Li X, Gu L, Xiu S, Bao X, Duan J, Zhang X (2015). MicroRNA-185 inhibits cell proliferation and induces cell apoptosis by targeting VEGFA directly in von Hippel-Lindau-inactivated clear cell renal cell carcinoma. Urol Oncol.

[48] Chen YS, Meng F, Li HL, Liu QH, Hou PF, Bai J, Zheng JN (2016). Dicer suppresses MMP-2-mediated invasion and VEGFA-induced angiogenesis and serves as a promising prognostic biomarker in human clear cell renal cell carcinoma. Oncotarget.

[49] Wang J, Wen J, Yi R, Liu F, Zhou J, Liu G, Li Q, Yang Z, Su X (2015). High selectivity of PI3 Kβ inhibitors in SETD2-mutated renal clear cell carcinoma. J BUON.

[50] Huang JL, Liao Y, Qiu MX, Li J, An Y (2017). Long non-coding RNA CCAT2 promotes cell proliferation and invasion through regulating Wnt/beta-catenin signaling pathway in clear cell renal cell carcinoma. Tumour Biol.

[51] Li M, Pei X, Wang G, Zhan J, Du J, Jiang H, Tang Y, Zhang H, He H (2017). Kindlin2 promotes clear cell renal cell carcinoma progression through the Wnt signaling pathway. Oncol Rep.

[52] Brooks SA, Brannon AR, Parker JS, Fisher JC, Sen O, Kattan MW, Hakimi AA, Hsieh JJ, Choueiri TK, Tamboli P, Maranchie JK, Hinds P, Miller CR, Nielsen ME, Rathmell WK (2014). ClearCode34: a prognostic risk predictor for localized clear cell renal cell carcinoma. Eur Urol.

[53] Rini BI, Escudier B, Martini JF (2018). Validation of the 16-gene recurrence score in patients with locoregional, high-risk renal cell carcinoma from a phase III trial of adjuvant sunitinib. Clin Cancer Res.

[54] Askeland EJ, Chehval VA, Askeland RW, Fosso PG, Sangale Z, Xu N, Rajamani S, Stone S, Brown JA (2015). Cell cycle progression score predicts metastatic progression of clear cell renal cell carcinoma after resection. Cancer Biomark..

