# Supplementary material for: Prognosis of clear cell renal cell carcinoma (ccRCC) based on a six-lncRNA-based risk score: an investigation based on RNA-sequencing data
Source: J Transl Med. 2019 Aug 23;17:281. doi: 10.1186/s12967-019-2032-y (PMC6708203; doi:10.1186/s12967-019-2032-y)
Supplement: Supplementary file 1 — Additional file 1: Table S1. Biological annotation of six prognostic lncRNAs. [file 12967_2019_2032_MOESM1_ESM.docx]

Table S1: Biological annotation of six prognostic lncRNAs

| **lncRNA** | **PMID** | **Ensembl ID** | **Transcript ID** | **Chromosome** | **Gene name** | **Gene type** | **Gene description** |
| --- | --- | --- | --- | --- | --- | --- | --- |
| **CTA-384D8.35** | **/** | ENSG00000272666 | ENST00000609178 | 22 | U62317.1 | lincRNA | novel transcript |
| **CTD-2263F21.1** | **/** | ENSG00000251257 | ENST00000510469 | 5 | AC010457.1 | antisense lncRNA | novel transcript |
|  |  | ENSG00000251257 | ENST00000510137 | 5 | AC010457.1 | antisense lncRNA | novel transcript |
| **LINC01510** | 30399588 | ENSG00000231210 | ENST00000650435 | 7 | LINC01510 | lincRNA | long intergenic non-protein coding RNA 1510 [Source:HGNC Symbol;Acc:HGNC:51196] |
|  | 30224058 | ENSG00000231210 | ENST00000450063 | 7 | LINC01510 | lincRNA | long intergenic non-protein coding RNA 1510 [Source:HGNC Symbol;Acc:HGNC:51196] |
|  | 29581707 | ENSG00000231210 | ENST00000441991 | 7 | LINC01510 | lincRNA | long intergenic non-protein coding RNA 1510 [Source:HGNC Symbol;Acc:HGNC:51196] |
|  |  | ENSG00000231210 | ENST00000458082 | 7 | LINC01510 | lincRNA | long intergenic non-protein coding RNA 1510 [Source:HGNC Symbol;Acc:HGNC:51196] |
| **RP11-352G9.1** | **/** | ENSG00000273009 | / | 3 | AC124944.8 | lincRNA | novel transcript |
| **RP11-395B7.2** | **/** | ENSG00000274993 | ENST00000610769 | 7 | AC254629.1 | antisense lncRNA | uncharacterized LOC105375431 [Source:NCBI gene;Acc:105375431] |
|  |  | ENSG00000274993 | ENST00000618276 | 7 | AC254629.1 | antisense lncRNA | uncharacterized LOC105375431 [Source:NCBI gene;Acc:105375431] |
| **RP11-426C22.4** | **/** | ENSG00000259807 | ENST00000566070 | 16 | AC009093.1 | lincRNA | novel transcript |
