# Supplementary material for: Prognosis of clear cell renal cell carcinoma (ccRCC) based on a six-lncRNA-based risk score: an investigation based on RNA-sequencing data
Source: J Transl Med. 2019 Aug 23;17:281. doi: 10.1186/s12967-019-2032-y (PMC6708203; doi:10.1186/s12967-019-2032-y)
Supplement: Supplementary file 2 — Additional file 2: Table S2. Gene Ontology (GO) analysis in the high-risk score group. [file 12967_2019_2032_MOESM2_ESM.docx]

**Table S2: Gene Ontology (GO) Annotation in high-risk score group**

| **Category** | **Term** | **Count** | ***P*-Value** | **Fold Enrichment** | **Bonferroni** | **Benjamini** | **FDR** |
| --- | --- | --- | --- | --- | --- | --- | --- |
| GOTERM_CC | GO:0005576~extracellular region | 224 | 3.71E-39 | 2.473671262 | 1.50E-36 | 1.50E-36 | 5.19E-36 |
| GOTERM_CC | GO:0005615~extracellular space | 160 | 3.49E-20 | 2.111894544 | 1.41E-17 | 7.05E-18 | 4.87E-17 |
| GOTERM_CC | GO:0005887~integral component of plasma membrane | 150 | 2.37E-14 | 1.884753943 | 9.60E-12 | 3.20E-12 | 3.32E-11 |
| GOTERM_MF | GO:0004252~serine-type endopeptidase activity | 45 | 3.60E-12 | 3.317371938 | 3.38E-09 | 3.38E-09 | 5.65E-09 |
| GOTERM_CC | GO:0005886~plasma membrane | 320 | 5.42E-11 | 1.380597889 | 2.19E-08 | 5.47E-09 | 7.57E-08 |
| GOTERM_BP | GO:0006955~immune response | 60 | 5.90E-11 | 2.545913984 | 1.91E-07 | 1.91E-07 | 1.07E-07 |
| GOTERM_CC | GO:0072562~blood microparticle | 32 | 3.11E-10 | 3.743055199 | 1.26E-07 | 2.51E-08 | 4.34E-07 |
| GOTERM_BP | GO:0007588~excretion | 16 | 4.30E-10 | 7.724899367 | 1.39E-06 | 6.95E-07 | 7.80E-07 |
| GOTERM_BP | GO:0006508~proteolysis | 65 | 4.32E-10 | 2.322297872 | 1.40E-06 | 4.66E-07 | 7.85E-07 |
| GOTERM_CC | GO:0016324~apical plasma membrane | 46 | 5.98E-10 | 2.810507082 | 2.42E-07 | 4.03E-08 | 8.35E-07 |
| GOTERM_BP | GO:0006958~complement activation, classical pathway | 25 | 7.12E-10 | 4.511068128 | 2.31E-06 | 5.76E-07 | 1.29E-06 |
| GOTERM_CC | GO:0009897~external side of plasma membrane | 37 | 2.79E-09 | 3.088459865 | 1.13E-06 | 1.61E-07 | 3.89E-06 |
| GOTERM_BP | GO:0050776~regulation of immune response | 32 | 1.49E-08 | 3.211475018 | 4.81E-05 | 9.61E-06 | 2.70E-05 |
| GOTERM_BP | GO:0044267~cellular protein metabolic process | 25 | 2.95E-08 | 3.784709701 | 9.54E-05 | 1.59E-05 | 5.35E-05 |
| GOTERM_BP | GO:0006956~complement activation | 21 | 4.88E-08 | 4.311958914 | 1.58E-04 | 2.26E-05 | 8.86E-05 |
| GOTERM_MF | GO:0003823~antigen binding | 22 | 8.71E-08 | 4.015200986 | 8.17E-05 | 4.09E-05 | 1.37E-04 |
| GOTERM_CC | GO:0016323~basolateral plasma membrane | 30 | 2.76E-07 | 2.963252033 | 1.12E-04 | 1.39E-05 | 3.86E-04 |
| GOTERM_BP | GO:0035725~sodium ion transmembrane transport | 18 | 4.02E-07 | 4.404779948 | 0.001301 | 1.63E-04 | 7.31E-04 |
| GOTERM_BP | GO:0006953~acute-phase response | 13 | 8.37E-07 | 5.954609929 | 0.002704 | 3.01E-04 | 0.00152 |
| GOTERM_MF | GO:0005102~receptor binding | 43 | 8.49E-07 | 2.289895077 | 7.96E-04 | 2.65E-04 | 0.00133 |
