# Supplementary material for: Prognosis of clear cell renal cell carcinoma (ccRCC) based on a six-lncRNA-based risk score: an investigation based on RNA-sequencing data
Source: J Transl Med. 2019 Aug 23;17:281. doi: 10.1186/s12967-019-2032-y (PMC6708203; doi:10.1186/s12967-019-2032-y)
Supplement: Supplementary file 3 — Additional file 3: Table S3. Gene Ontology (GO) analysis in the low-risk score group. [file 12967_2019_2032_MOESM3_ESM.docx]

**Table S3: Gene Ontology (GO) Annotation in low-risk score group**

| **Category** | **Term** | **Count** | ***P*-Value** | **Fold Enrichment** | **Bonferroni** | **Benjamini** | **FDR** |
| --- | --- | --- | --- | --- | --- | --- | --- |
| GOTERM_CC | GO:0005576~extracellular region | 144 | 2.25E-26 | 2.5468323 | 7.46E-24 | 7.46E-24 | 3.05E-23 |
| GOTERM_CC | GO:0005615~extracellular space | 114 | 8.81E-19 | 2.4099109 | 2.92E-16 | 1.46E-16 | 1.20E-15 |
| GOTERM_BP | GO:0006814~sodium ion transport | 17 | 1.40E-08 | 6.0658295 | 3.51E-05 | 3.51E-05 | 2.47E-05 |
| GOTERM_CC | GO:0005887~integral component of plasma membrane | 89 | 8.56E-08 | 1.7910071 | 2.84E-05 | 9.47E-06 | 1.16E-04 |
| GOTERM_BP | GO:0007588~excretion | 11 | 3.36E-07 | 8.5924548 | 8.42E-04 | 4.21E-04 | 5.92E-04 |
| GOTERM_MF | GO:0004252~serine-type endopeptidase activity | 27 | 5.06E-07 | 3.146831 | 3.68E-04 | 3.68E-04 | 7.67E-04 |
| GOTERM_BP | GO:0006953~acute-phase response | 11 | 5.76E-07 | 8.1518161 | 0.0014435 | 4.81E-04 | 0.0010157 |
| GOTERM_BP | GO:0006508~proteolysis | 41 | 7.47E-07 | 2.3699552 | 0.001873 | 4.69E-04 | 0.0013183 |
| GOTERM_CC | GO:0072562~blood microparticle | 19 | 6.51E-06 | 3.559375 | 0.0021593 | 5.40E-04 | 0.0088345 |
| GOTERM_CC | GO:0005578~proteinaceous extracellular matrix | 26 | 9.11E-06 | 2.7625 | 0.0030191 | 6.05E-04 | 0.0123575 |
| GOTERM_MF | GO:0005215~transporter activity | 21 | 1.61E-05 | 3.0897103 | 0.0116398 | 0.005837 | 0.0243779 |
| GOTERM_BP | GO:0035725~sodium ion transmembrane transport | 12 | 3.87E-05 | 4.7509962 | 0.0925619 | 0.0192385 | 0.0682735 |
| GOTERM_CC | GO:0016324~apical plasma membrane | 25 | 9.96E-05 | 2.4463058 | 0.0325204 | 0.005495 | 0.1350343 |
| GOTERM_MF | GO:0005125~cytokine activity | 17 | 2.99E-04 | 2.8706886 | 0.195401 | 0.0699068 | 0.4517112 |
| GOTERM_MF | GO:0008201~heparin binding | 16 | 3.29E-04 | 2.972007 | 0.212746 | 0.0580481 | 0.4968774 |
| GOTERM_BP | GO:0055078~sodium ion homeostasis | 5 | 3.83E-04 | 13.137224 | 0.6176469 | 0.1480565 | 0.6737353 |
| GOTERM_BP | GO:0009813~flavonoid biosynthetic process | 6 | 4.87E-04 | 8.670568 | 0.7053312 | 0.1601715 | 0.8554999 |
| GOTERM_CC | GO:0031225~anchored component of membrane | 13 | 6.09E-04 | 3.275885 | 0.1831277 | 0.0284826 | 0.8233136 |
| GOTERM_CC | GO:0016323~basolateral plasma membrane | 17 | 6.17E-04 | 2.6893056 | 0.1851614 | 0.0252709 | 0.8334178 |
| GOTERM_BP | GO:0052696~flavonoid glucuronidation | 6 | 7.81E-04 | 7.8823345 | 0.8591995 | 0.2173357 | 1.3690041 |
