# Supplementary material for: Prognosis of clear cell renal cell carcinoma (ccRCC) based on a six-lncRNA-based risk score: an investigation based on RNA-sequencing data
Source: J Transl Med. 2019 Aug 23;17:281. doi: 10.1186/s12967-019-2032-y (PMC6708203; doi:10.1186/s12967-019-2032-y)
Supplement: Supplementary file 4 — Additional file 4: Table S4. Kyoto Encyclopedia of Genes and Genomes (KEGG) pathway analysis in the high-risk score group. [file 12967_2019_2032_MOESM4_ESM.docx]

**Table S4: Kyoto Encyclopedia of Genes and Genomes (KEGG) pathways in high-risk score group**

| **Term** | **Count** | ***P*-Value** | **Fold Enrichment** | **Bonferroni** | **Benjamini** | **FDR** |
| --- | --- | --- | --- | --- | --- | --- |
| hsa05322:Systemic lupus erythematosus | 25 | 2.60E-07 | 3.3485172 | 6.48E-05 | 6.48E-05 | 3.38E-04 |
| hsa04080:Neuroactive ligand-receptor interaction | 35 | 8.96E-06 | 2.2678044 | 0.0022283 | 0.0011148 | 0.0116305 |
| hsa04610:Complement and coagulation cascades | 14 | 8.83E-05 | 3.6416337 | 0.02174 | 0.0072998 | 0.1145363 |
| hsa00980:Metabolism of xenobiotics by cytochrome P450 | 14 | 1.86E-04 | 3.3955774 | 0.0451529 | 0.0114845 | 0.2406161 |
| hsa04060:Cytokine-cytokine receptor interaction | 27 | 3.98E-04 | 2.1069452 | 0.0943564 | 0.0196267 | 0.5154201 |
| hsa04966:Collecting duct acid secretion | 8 | 5.22E-04 | 5.3179413 | 0.1218631 | 0.0214259 | 0.6752787 |
| hsa05034:Alcoholism | 22 | 7.61E-04 | 2.2308313 | 0.1726446 | 0.0267112 | 0.9832887 |
| hsa00982:Drug metabolism - cytochrome P450 | 12 | 0.0011795 | 3.1673033 | 0.2546322 | 0.0360681 | 1.5205865 |
| hsa05204:Chemical carcinogenesis | 13 | 0.001416 | 2.9165584 | 0.2973027 | 0.0384447 | 1.8228195 |
| hsa04971:Gastric acid secretion | 12 | 0.0021341 | 2.9503647 | 0.4125434 | 0.0518052 | 2.7354718 |
| hsa00350:Tyrosine metabolism | 8 | 0.0026896 | 4.1024119 | 0.4886011 | 0.059143 | 3.4360892 |
| hsa00830:Retinol metabolism | 11 | 0.0028721 | 3.0373626 | 0.5113892 | 0.0579363 | 3.6653217 |
| hsa04960:Aldosterone-regulated sodium reabsorption | 8 | 0.005081 | 3.6816517 | 0.7187197 | 0.0929607 | 6.3994647 |
| hsa04514:Cell adhesion molecules (CAMs) | 17 | 0.0052556 | 2.1487104 | 0.7307455 | 0.0894635 | 6.6124644 |
| hsa05033:Nicotine addiction | 8 | 0.005872 | 3.5896104 | 0.7692536 | 0.0931357 | 7.3609494 |
