# Supplementary material for: Prognosis of clear cell renal cell carcinoma (ccRCC) based on a six-lncRNA-based risk score: an investigation based on RNA-sequencing data
Source: J Transl Med. 2019 Aug 23;17:281. doi: 10.1186/s12967-019-2032-y (PMC6708203; doi:10.1186/s12967-019-2032-y)
Supplement: Supplementary file 5 — Additional file 5: Table S5. Kyoto Encyclopedia of Genes and Genomes (KEGG) pathway analysis in the low-risk score group. [file 12967_2019_2032_MOESM5_ESM.docx]

**Table S5: Kyoto Encyclopedia of Genes and Genomes (KEGG) pathways in low-risk score group**

| **Term** | **Count** | ***P*-Value** | **Fold Enrichment** | **Bonferroni** | **Benjamini** | **FDR** |
| --- | --- | --- | --- | --- | --- | --- |
| hsa04610:Complement and coagulation cascades | 15 | 1.14E-07 | 6.0086957 | 2.64E-05 | 2.64E-05 | 1.46E-04 |
| hsa00980:Metabolism of xenobiotics by cytochrome P450 | 14 | 1.86E-06 | 5.2291892 | 4.31E-04 | 2.16E-04 | 0.002388 |
| hsa00982:Drug metabolism - cytochrome P450 | 13 | 4.45E-06 | 5.2841176 | 0.0010311 | 3.44E-04 | 0.0057084 |
| hsa05204:Chemical carcinogenesis | 13 | 2.50E-05 | 4.4915 | 0.0057934 | 0.0014515 | 0.0321467 |
| hsa00830:Retinol metabolism | 11 | 9.61E-05 | 4.6775385 | 0.0220453 | 0.0044485 | 0.1232804 |
| hsa04080:Neuroactive ligand-receptor interaction | 24 | 1.47E-04 | 2.3948014 | 0.0334583 | 0.0056558 | 0.1881386 |
| hsa04976:Bile secretion | 11 | 1.61E-04 | 4.4063768 | 0.0366199 | 0.0053154 | 0.2062334 |
| hsa00140:Steroid hormone biosynthesis | 9 | 0.0010219 | 4.2889655 | 0.2111633 | 0.0292143 | 1.3039898 |
| hsa00983:Drug metabolism - other enzymes | 8 | 0.0011654 | 4.8069565 | 0.2370331 | 0.0296128 | 1.485935 |
| hsa00053:Ascorbate and aldarate metabolism | 6 | 0.0024577 | 6.1422222 | 0.4349746 | 0.0554895 | 3.1097152 |
| hsa00860:Porphyrin and chlorophyll metabolism | 7 | 0.0036362 | 4.6066667 | 0.5704982 | 0.0739527 | 4.5690035 |
| hsa04971:Gastric acid secretion | 9 | 0.0045079 | 3.4076712 | 0.6494267 | 0.0836426 | 5.6353109 |
| hsa04020:Calcium signaling pathway | 15 | 0.0050707 | 2.3162011 | 0.6925333 | 0.0867286 | 6.3179509 |
| hsa00350:Tyrosine metabolism | 6 | 0.0078205 | 4.7382857 | 0.8382142 | 0.1219975 | 9.588163 |
| hsa00040:Pentose and glucuronate interconversions | 6 | 0.0088211 | 4.6066667 | 0.8719801 | 0.1280629 | 10.751773 |
