# Supplementary figures and images for: Prognosis of clear cell renal cell carcinoma (ccRCC) based on a six-lncRNA-based risk score: an investigation based on RNA-sequencing data
Source: J Transl Med. 2019 Aug 23;17:281. doi: 10.1186/s12967-019-2032-y (PMC6708203; doi:10.1186/s12967-019-2032-y)

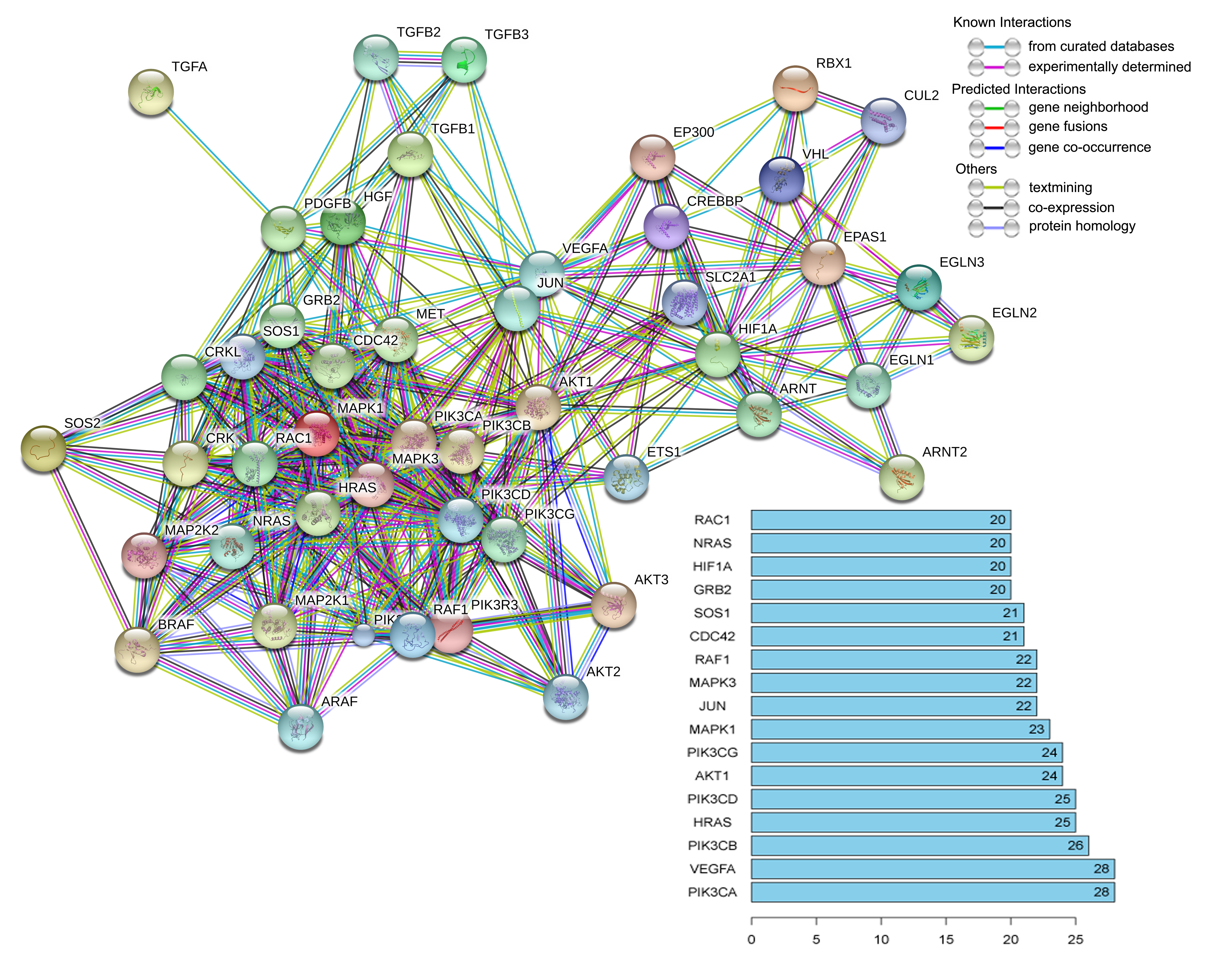

Supplement: Supplementary file 6 — Additional file 6: Fig. S1. Protein–protein interaction (PPI) network of genes from the ‘Renal cell carcinoma pathway. [file 12967_2019_2032_MOESM6_ESM.tif]

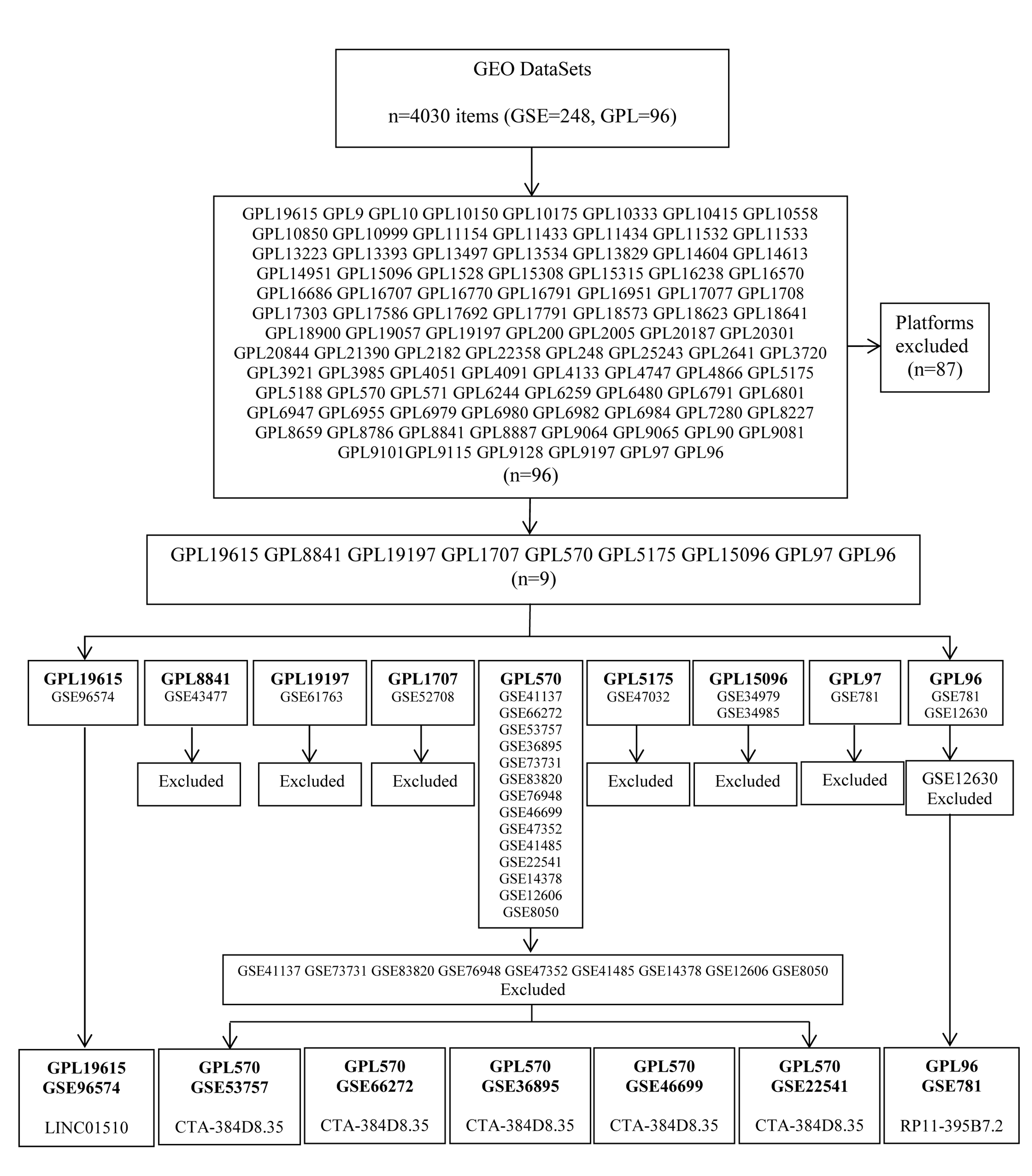

Supplement: Supplementary file 7 — Additional file 7: Fig. S2. Flowchart for lncRNA validation in clear cell renal cell carcinoma (ccRCC) based on Gene Expression Omnibus (GEO) data. [file 12967_2019_2032_MOESM7_ESM.tif]

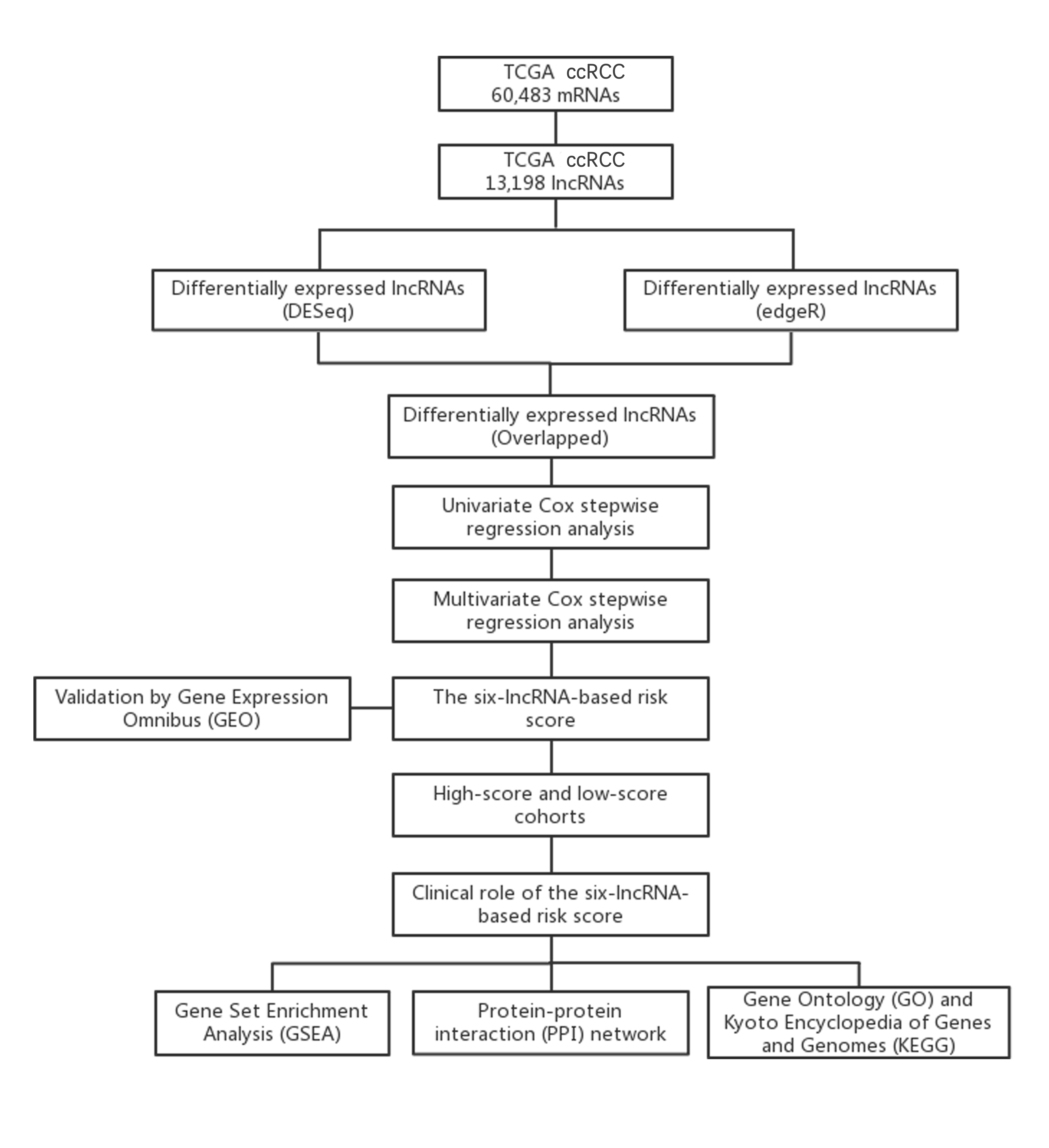

Supplement: Supplementary file 8 — Additional file 8: Fig. S3. Flow chart summarizing the current study. [file 12967_2019_2032_MOESM8_ESM.tif]

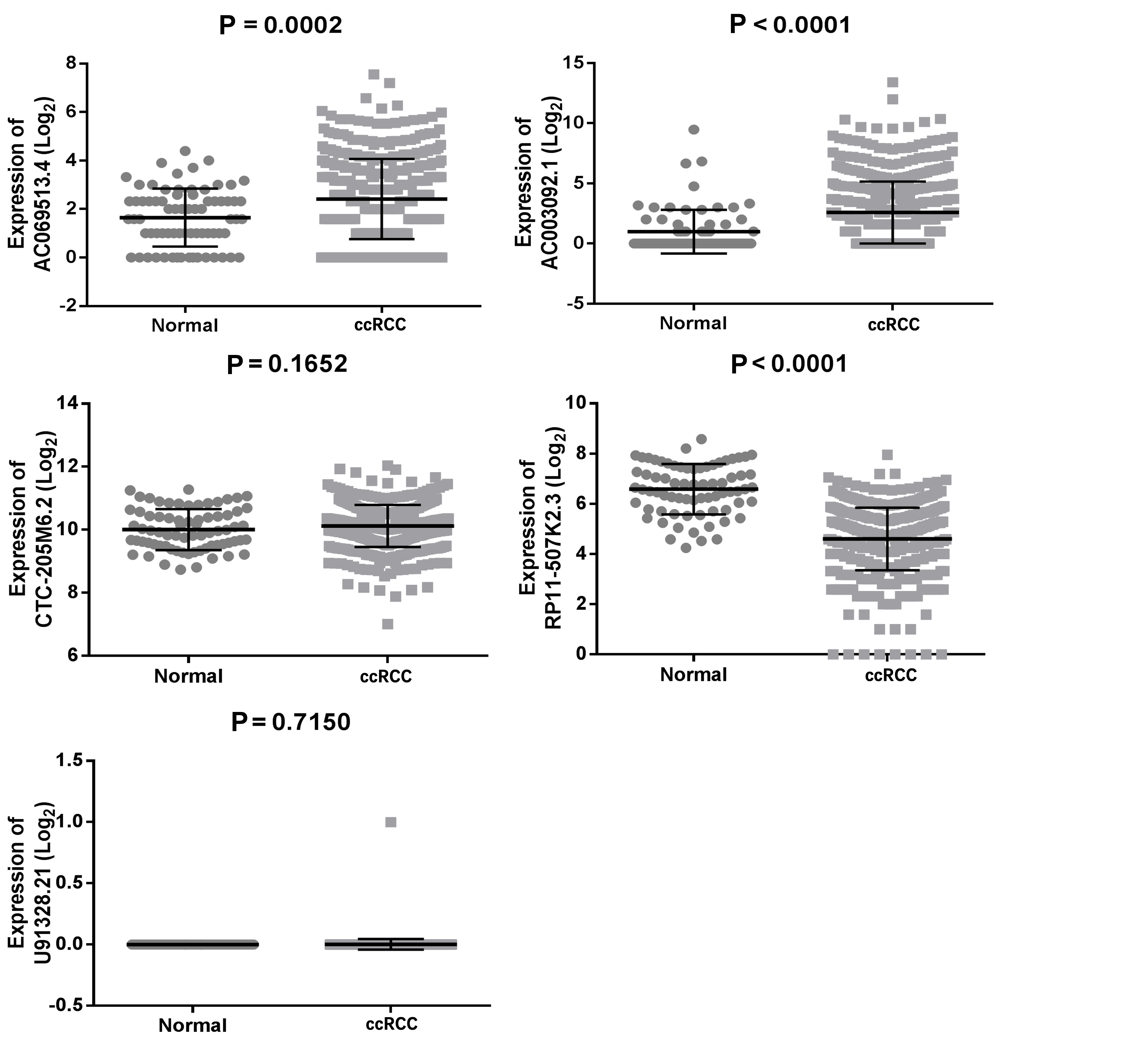

Supplement: Supplementary file 9 — Additional file 9: Fig. S4. Differential expression of the five identified lncRNAs between clear cell renal cell carcinoma (ccRCC) and para-tumorous renal tissues in the study by Shi et al. [file 12967_2019_2032_MOESM9_ESM.tif]
